# Modulating the direction of catalytic glyoximate sites of covalent organic frameworks towards electrocatalytic nitrate reduction

**DOI:** 10.1039/d5sc02151k

**Published:** 2025-07-21

**Authors:** Shuai Yang, Shuai Bi, Lipeng Zhai, Qing Xu

**Affiliations:** a CAS Key Laboratory of Low-Carbon Conversion Science and Engineering, Shanghai Advanced Research Institute, Chinese Academy of Sciences Shanghai 201210 P. R. China xuqing@sari.ac.cn; b Department of Chemistry, City University of Hong Kong Kowloon Hong Kong 999077 P. R. China; c Henan Key Laboratory of Functional Salt Materials, Center for Advanced Materials Research, Zhongyuan University of Technology Zhengzhou Henan 450007 P. R. China zhailp@zut.edu.cn; d School of Chemical Engineering, University of Chinese Academy of Sciences Beijing 100049 P.R. China

## Abstract

Two-dimensional (2D) covalent organic frameworks (COFs) with metal centers are ideal templates to construct electrocatalysts due to their high degree of structural controllability. However, the metal centers are stacked in columns with limited space, which impedes the mass delivered to catalytic sites across the pore channels. Herein, we demonstrate a topologic synthesis strategy for constructing catalytic sites in three-dimensional (3D) space. The designed 3D COF adopts an *ffc* topology, with a large space of 1.15 and 1.53 nm between the metal sites along the parallel and vertical directions, respectively. *In situ* spectroscopy revealed that ∼100% Ni–N_4_ sites in 3D frameworks were reconstructed to Ni–N_4_–NO, while the reconstruction proportion of Ni–N_4_ sites was ∼40% for 2D COF (with a distance of 0.38 nm between metal sites). The catalytic 3D COFs enable the electrochemical synthesis of NH_3_*via* the reduction of nitrate (NO_3_RR) at a rate of 9.51 mg mg_cat_^−1^ h^−1^, corresponding to 140% of that for the 2D COF at −0.7 V *vs.* RHE. Theoretical calculations further revealed that the reconstructed Ni–N_4_–NO site had a stronger binding ability of the reactants and intermediates than that of the initial Ni–N_4_ site and thus contributed to higher activity. This work provides general design strategies for heterogeneous catalysts in electrocatalytic systems.

## Abstract 2

The two novel COFs in 2D and 3D topologies were constructed for catalyzing the NO_3_^−^ reduction reaction, and the 3D COF showed hihger activity than 2D COFs.

## Graphical abstract

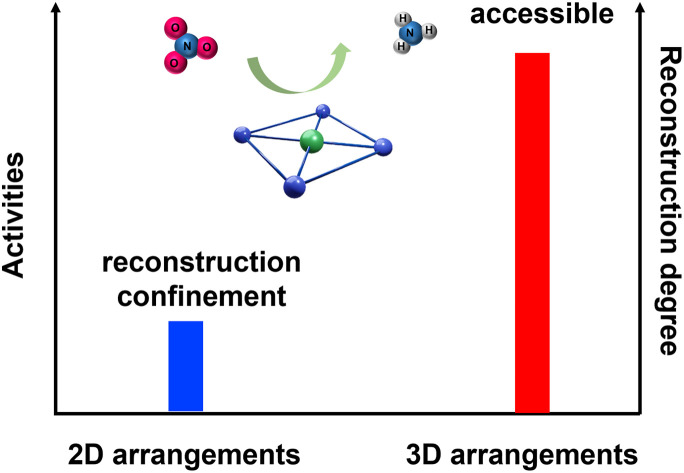

## Introduction

Covalent organic frameworks (COFs) are emerging porous crystalline polymers constructed using steerable building blocks *via* covalent polymerization.^1–10^ Their high porosities, conjugation, precise atomic-level manipulability, and structural diversity have led to their use in numerous cutting-edge applications, including gas separation and adsorption,^11–13^ electro- and photo-catalysis,^14–25^ and energy conversion.^26–30^ By using metal porphyrin/phthalocyanin-based knots, COFs have been widely employed as electrocatalysts in oxygen reduction reactions,^31–36^ oxygen evolution reactions,^33,37–39^ and CO_2_ reduction reactions.^40–45^ To modulate the catalytic properties, different linkers and linkages have been integrated into the frameworks, resulting in the modulation of the electronic states of metal sites because of the charge transfer between the building blocks.^31–45^ However, most of these catalytic COFs adopt a 2D topology; the stacking interaction in 2D COFs causes the metal centers to be arranged into a column, which results in a narrow distance (<0.4 nm) between metal sites, hindering the reactants' and the intermediates' access to the metal sites. More importantly, *in situ* technology development has demonstrated the dynamic chemistry of catalytic sites and their ability to reconstruct efficient active sites. The space-limited 2D stacking of the catalytic sites is negative on the reconstruction and mass access, which drew our attention to the 3D structures.

The NO_3_^−^ reduction reaction (NO_3_RR) for NH_3_ production is a promising alternative to the Haber–Bosch process.^46–50^ NO_3_RR is a complicated reaction that involves the transfer of multi-electron–proton transfer and faces competition from various products and pathways.^48,49,51^ Many efforts have been devoted to developing catalysts for NO_3_RR, such as noble-metal-based materials,^52,53^ alloys,^53–56^ metallic compounds,^57–59^ and single-metal-atom catalysts.^57,60,61^ However, most of these materials are composed of heterogeneous active species with random arrangements, leading to confusing structure–activity relationships. COFs have provided us with a new opportunity to construct highly efficient catalysts for NO_3_RR due to their well-defined sites and porosities. Until now, there have been few reports for the catalysis of NO_3_RR using 2D COFs.^62^ However, the dynamic changes of metal sites in 2D COFs are rarely observed. Studying the changes of metal sites is important to gain a better understanding of the multi-electron process of NO_3_RR, which will guide our design of highly efficient catalysts in complicated reactions.

Herein, we sought to construct 3D COFs to study the influence of directions and spaces between metal sites on NO_3_RR, in which the metal sites (Ni–N_4_) were arranged in an *ffc* topology and adopted parallel and vertical directions. The different directions of extended frameworks resulted in distances between the close metal sites in parallel and vertical directions of 1.15 and 1.53 nm, respectively, which were ∼3 and ∼4 times that of the control COF (0.38 nm). The large space in 3D COFs not only benefited accessing the reactants and intermediates but also promoted the initial Ni–N_4_ sites being reconstructed into Ni–N_4_–NO, thus contributing to a higher activity compared to the 2D COF with the same metal sites.

## Results and discussion

**Fig. 1:**
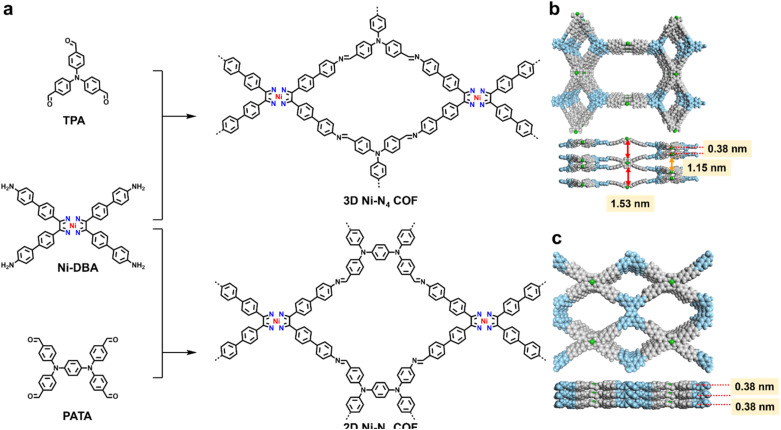
(a) Design and synthesis of catalytic COFs with topologic modulation for NO_3_RR. (b) The distances between the metal sites in 3D Ni–N_4_-COF. (c) The distances between the Ni–N_4_ sites in 2D Ni–N_4_-COF. The water molecules around the Ni sites are omitted for clarity.

We synthesized the new 3D COF (3D-NiN_4_-COF) from nickel-coordinated glyoximate di-biphenyl amine (Ni-DBA) and tris(4-aminophenyl)amine (TPA) in 1,2-dichlorobenzene/propane-2-ol/acetic acid at 120 °C for 5 days, obtaining a yield of 75% (Fig. 1a). As a control, we have synthesized 2D Ni–N_4_-COF by using *N*,*N*,*N*′,*N*′-tetrakis(4-aminophenyl)-1,4-benzenediamine (PATA) as the substituted linkers in the same condition, with a corresponding yield of 81% (Fig. 1a).

**Fig. 2:**
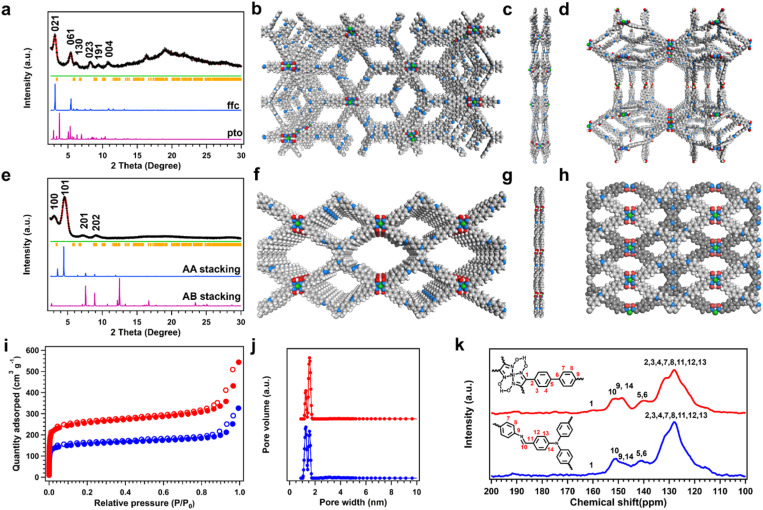
PXRD patterns of (a) 3D-Ni-N_4_-COF, comparison between the experimental profiles (black crosses), Pawley refined profiles (red line), the simulated patterns for *ffc* net (blue line), *pto* net (violet line), the Bragg positions (yellow bars), and the refinement differences (green line). (b) Top view and (c) side view of *ffc* topological 3D-Ni-N_4_-COF. (d) Top view of *pto* topological 3D-Ni-N_4_-COF. PXRD patterns of (e) 2D-Ni-N_4_-COF; comparison between the experimental profiles (black cross), Pawley refined profiles (red line), the simulated patterns for eclipsed (AA) stacking mode (blue line), (AB) stacking mode (violet line), the Bragg positions (yellow bar), and the refinement differences (green line). (f) Top view and (g) side view of AA stacking 2D-Ni-N_4_-COF. (h) Top view of AB stacking 2D-Ni-N_4_-COF. (i) Nitrogen sorption experiments (77 K), (j) the pore size distribution and (k) solid-state CP/MAS ^13^C NMR spectra of 2D-Ni-N_4_-COF (blue) and 3D-Ni-N_4_-COF (red), respectively.

Powder X-ray diffractometry (PXRD) was initially used to determine the crystallinity and topology of the synthesized COFs. The Ni-DBA is a typical 4-connected building unit monomer that maintained the planar atomic skeleton, and the TPA unit is a non-planar 3-connected organic block.^63,64^ The topological analysis initially proceeded on the prepared COFs for recognizing the 3D structures. Combined with the crystal face indexing, we established a CMCM (Schoenflies: D2H-17) orthorhombic space group, which was the *ffc* topology with a two-fold interpenetration structure (Fig. 2a–c, Table S1). The optimized *ffc* topology crystal was executed in progress to obtain diffraction peaks that matched well with the experiment curve. The PXRD peaks at 3.10°, 5.36°, 6.24°, 8.22°, 9.36°, and 10.80° correspond to the (021), (061), (130), (023), (191), and (004) facets, respectively (Fig. 2a, black cross). The experiment data fit well with the refined pattern for the 2-fold interpenetrated *ffc* topology, showing that the refined patterns (red curve) are in accordance with the PXRD patterns (black crosses), with small differences (green curve) observed (Fig. 2a, refined parameters: *R*_wp_ = 4.63%, *R*_p_ = 3.60%). Thus, the crystallographic data confirmed the existence of extended 3D Ni–N_4_ frameworks. The open spaces between Ni–N_4_ sites in the simulated 3D COF were 1.15 and 1.53 nm between the metal sites in parallel and vertical directions (Fig. 1b). Moreover, *pto* and other topologies are also possible owing to the four-connected planar linkers and three-connected triangular non-planar linkers, but the optimized crystal did not match the experiments' PXRD curve (Fig. 2a and d, S1, S2 and Table S2).

The 2D-NiN_4_-COF also displayed good crystallinity. The PXRD pattern for 2D-NiN_4_-COF displayed peaks at 3.08°, 4.56°, 7.20°, and 9.08°, which could be attributable to the (100), (101), (201), and (202) facets of the COF, respectively (Fig. 2e, black cross). The eclipsed (AA, blue curve) and staggered (AB, violet curve) stacking models were simulated, respectively (Fig. 2e–h, S3, Tables S3 and S4).^18,65^ The experimental PXRD pattern of the COF well matched the simulated PXRD pattern of the aligned AA stacking model with *R*_wp_ and *R*_p_ parameters of 3.42% and 2.74%, respectively, and the corresponding unit cell of *a* = 32.9727 Å, *b* = 24.8991 Å, *c* = 3.7971 Å, and *α* = *β* = *γ* = 90° with the space group of P1 (Fig. 2f and g). Accordingly, the distances between the Ni–N_4_ sites in the frameworks were 0.38 nm (Fig. 1c and 2g).

N_2_-sorption experiments were conducted to assess the porosities of 2D-Ni-N_4_-COF and 3D-Ni-N_4_-COF. Both COFs displayed type I sorption isotherms, consistent with both frameworks possessing luxuriant micropore structures (Fig. 2i and j). 3D-Ni-N_4_-COF exhibited a higher Brunauer–Emmett–Teller (BET) specific surface area of 842 m^2^ g^−1^ than that of 2D-Ni-N_4_-COF (512 m^2^ g^−1^), with the pore volumes of 1.45 and 0.61 cm^3^ g^−1^, respectively (Table S5). The pore size distribution curves showed the 3D COF had a pore size of 1.55 nm, larger than that of the 2D COF (Fig. 2j).

Chemical structures were examined using Fourier-transform infrared (FT-IR) and ^13^C solid-state nuclear magnetic resonance (NMR) spectroscopies. The FT-IR spectra showed the C=O vibrational peak at 1698 cm^−1^ of two formyl group-containing monomers. After constructing 2D and 3D COFs, the new peaks were observed at 1678 and 1625 cm^−1^ that correspond to C=N units (Fig. S4). This phenomenon verified imine linkages between the –CHO and –NH_2_ moieties in the monomers *via* Schiff-base chemistry. Moreover, the formation of two types of frameworks was validated using ^13^C solid-state NMR spectroscopy, with the peaks at 151 ppm in the spectrum of two COFs attributable to imine linkages (Fig. 2k).^18^ The peaks from all the carbon atoms in both COFs were also observed, further confirming the successful synthesis of COFs.

The morphologies of the two COFs were examined using scanning electron microscopy and transmission electron microscopy (Fig. S5–10). Fig. S5 and S6 display clear differences between 2D-Ni-N_4_-COF and 3D-Ni-N_4_-COF. The two COFs exhibit nano-coral-like morphologies composed of stacked blocks (pillars for the 2D COF and balls for the 3D COF). The 3D COF also exhibits clear lattice fringes from which a ten-interval distance of 25.7 nm was measured; hence, the single-interval distance of 2.57 nm corresponds to the interplanar spacing of the (040) facet (Fig. S10). The thermal stability was also assessed by thermogravimetry analysis (TGA), and the results showed that both COFs began to decompose after ∼200 °C, suggesting their similar thermal stability (Fig. S11).

**Fig. 3:**
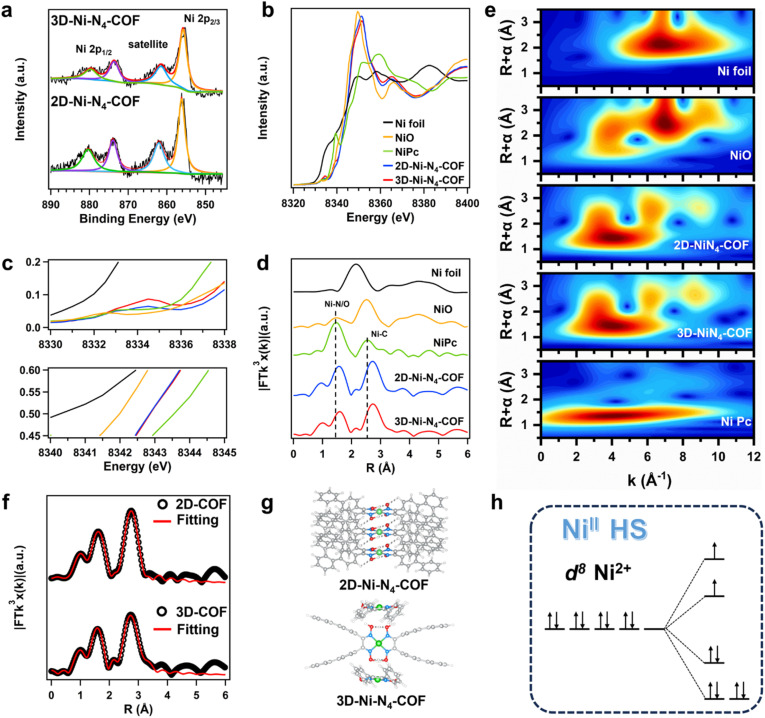
(a) High-resolution XPS spectra of the Ni 2p region of 2D-Ni-N_4_-COF (down) and 3D-Ni-N_4_-COF (up). (b) Complete and (c) partially enlarged Ni K-edge XANES pattern of Ni foil (black), NiO (yellow), NiPc (green), 2D-Ni-N_4_-COF (blue), and 3D-Ni-N_4_-COF (red). (d) Ni K-edge *k*^3^-weighted Fourier transform spectra from EXAFS, the same colors as that of (b). (e) Wavelet transform contour spectra for the Ni K-edge *k*^2^-weighted EXAFS data. (f) Ni K-edge *k*^3^-weighted Fourier transform spectra from EXAFS of 2D-Ni-N_4_-COF (up) and 3D-Ni-N_4_-COF (down). The experimental results are represented by black dots, while the red line indicates the best–fit curves for *R* = 1–3.0 Å, using corresponding *k*^2^*χ*(*k*) functions in *k* = 3–11 Å^−1^. (g) Corresponding configurations of 2D-Ni-N_4_-COF and 3D-Ni-N_4_-COF. (h) 3d electron configuration of Ni centers in two COFs.

The chemical compositions of the two COFs were investigated using X-ray photoelectron spectroscopy (XPS). The survey spectrum of 2D-Ni-N_4_-COF revealed the presence of C, N, O, and Ni with contents of 83.8%, 7.2%, 4.7%, and 4.3%, respectively. Similarly, 3D-Ni-N_4_-COF was found to contain 82.5%, 7.1%, 5.4%, and 5.0% of C, N, O, and Ni, respectively (Table S6). High-resolution N 1s spectra demonstrated that the chemical components were similar and agreed with the expected structures (Fig. S12 and S13). The high-resolution Ni 2p spectra show doublets at 874 and ∼856 eV that correspond to the nickel (II) glyoximate units (Fig. 3a). The intensity of the satellite peak in each spectrum provides a qualitative assessment of the unpaired-electron density in the hybridized 3d orbital and is consistent with Ni centers in high-spin states.^66,67^

X-ray absorption fine structure (XAFS) spectroscopy was performed to describe the electronic states and coordination environments of the 2D and 3D COFs. Ni K-edge X-ray absorption near edge structure (XANES) revealed a strong white line peak attributable to the transition of an electron from the occupied 1s core orbital to an unoccupied 4p orbital (Fig. 3b and c). Moreover, the distinct wide pre-edge peaks are attributable to a quadrupole 1s → 3d transition resulting from 3d orbital holes associated with (+2) Ni. The coordination configurations of the nickel glyoximate were explored using extended XAFS (EXAFS) spectroscopy (Fig. 3d). The Fourier-transformed (FT) *k*^3^-weighted EXAFS spectra of the 2D and 3D COFs show peaks at 1.6 and 2.7 Å, respectively; such distinguishing peaks exclude the presence of metal oxides and metal clusters, as Ni foil exhibits a Ni–Ni bond length of 2.1 Å, and the Ni–O–Ni path is 2.5 Å long based on standard-sample data. Here, the FT *k*^3^-weighted EXAFS peak at 2.7 Å is wide and matches the high-shell Ni–C peak observed for nickel phthalocyanine (NiPc).

To clarify the origin of the wide FT-EXAFS peak, we examined the wavelet-transformed (WT) K-edge EXAFS oscillation based on its ability to simultaneously resolve and separate backscattering atoms in *k*-space and radial distance. The intensity maximum of each sample exhibited different coordinates (*k*, *R*), whose locations are primarily associated with the atomic-number-dependent scattering function. Fig. 2e reveals that the 2D and 3D COFs show identical images; the three centers located at 4.1, 6.1, and 8.63 Å^−1^ are consistent with Ni–N/O, Ni–C, and Ni–Ni units, respectively. Therefore, we determined the Ni-atom coordination shell, with atomic lengths and coordination numbers further obtained by Feff fitting (Fig. 2f and Table S7). The fitted FT-EXAFS spectra reveal the presence of Ni–N, Ni–Ni, and Ni–C bonds in the 2D and 3D COFs, consistent with the refined XRD data (Fig. 3g). The Ni center 3d orbital electron states were also determined to be the high spin states.

**Fig. 4:**
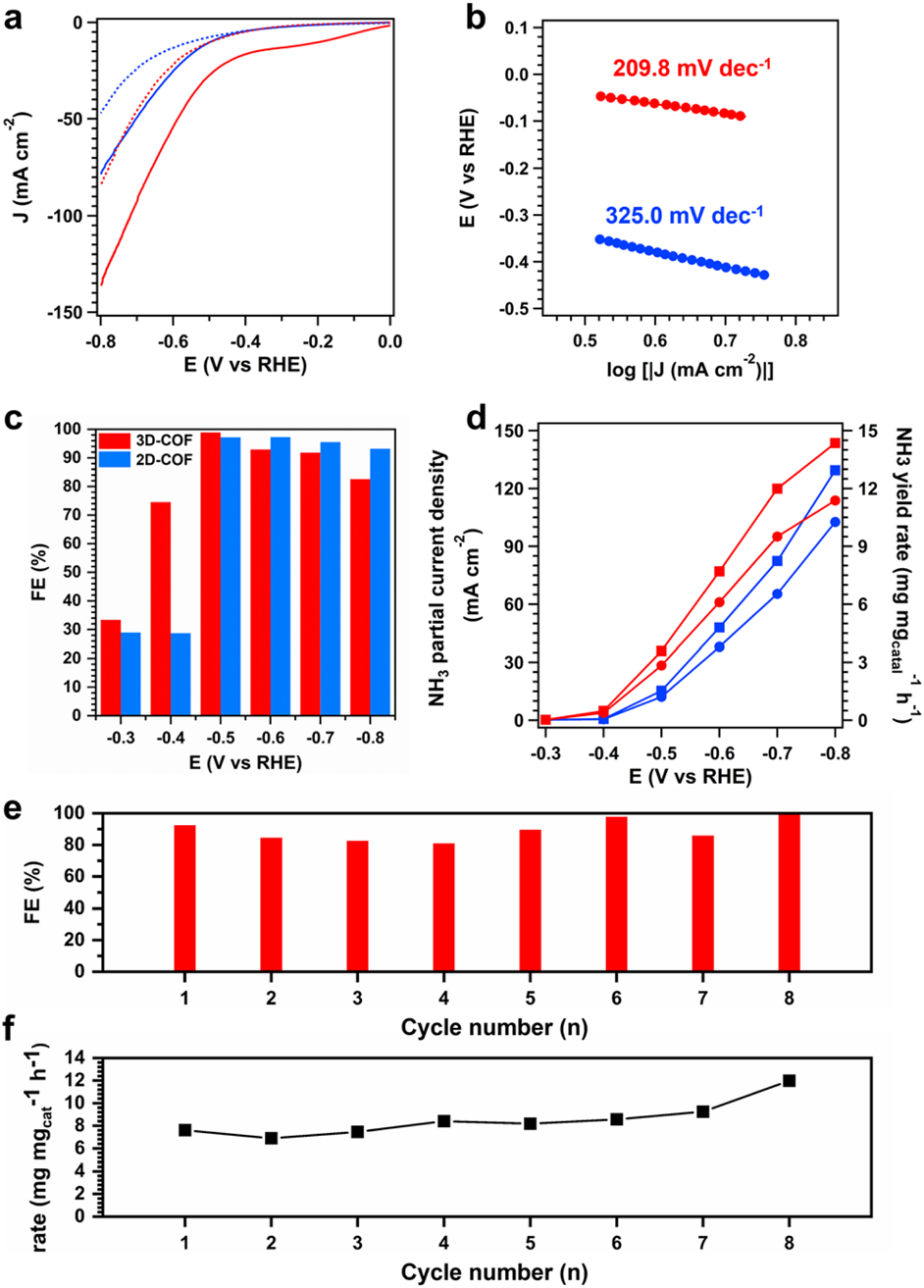
NO_3_RR activity and selectivity measurements. (a) Linear sweep voltammetry curves of 2D Ni–N_4_-COF (blue) and 3D Ni–N_4_-COF (red) were tested in 1 M KOH (dashed line) and 1 M KOH that contained 0.5 M KNO_3_ (solid line), respectively. (b) The LSV-derived Tafel slopes of 2D Ni–N_4_–COF (blue) and 3D Ni–N_4_-COF (red) in 1 M KOH that contained 0.5 M KNO_3_, respectively. (c) NH_3_ FE of 2D Ni–N_4_-COF (blue) and 3D Ni–N_4_-COF (red) at each given potential. (d) NH_3_ yield rate and partial current density of 2D Ni–N_4_-COF (blue) and 3D Ni–N_4_-COF (red). Cycling test of 3D Ni–N_4_-COF at −0.6 V showing the (e) average NH_3_ faradaic efficiency and (f) production rate within each cycle.

Electrochemical nitrate reduction was evaluated using a customized H-cell fitted with a three-electrode system under Ar-saturated conditions. We prepared working electrodes by dropping inks of COF powders dispersed in solutions of ethanol and Nafion onto carbon paper. The catalytic activities of 2D-Ni-N_4_-COF and 3D-Ni-N_4_-COF were explored using linear sweep voltammetry in electrolytes with and without KNO_3_ (Fig. 4a). Higher current densities were clearly observed for the KNO_3_-containing electrolyte, as well as for the COF with the 3D Ni–N_4_ arrangement. 3D-Ni–N_4_-COF exhibited an onset potential (*E*_o_) of only −0.19 V *vs.* RHE in the KNO_3_-containing electrolyte, which is lower than that exhibited by 2D-Ni-N_4_-COF (−0.50 V). 3D-Ni-N_4_-COF also exhibited a lower Tafel slope than 2D-Ni-N_4_-COF (209.8 *vs.* 325.0 mV dec^−1^, respectively), indicative of faster electron transfer during the reduction of NO_3_^−^ (Fig. 4b).

Product selectivity was subsequently evaluated by chronoamperometry for 1 h and spectrophotometrically quantified using the indophenol blue method (Fig. S14–16). While both COFs maintained faradaic efficiencies (FEs) of >90% over a wide range (−0.5 to −0.7 V), they showed different activities, with 3D-Ni-N_4_-COF exhibiting an average current density that ranged between 35.8 and 143.5 mA cm^−2^ as the potential was varied from −0.5 to −0.7 V, while that for 2D-Ni-N_4_-COF ranged from 15.3 to 129.4 mA cm^−2^ (Fig. 4c and d). We also calculated the corresponding productivities, which ranged between 2.84 and 9.51 mg mg_cat_^−1^ h^−1^ for 3D-Ni-N_4_-COF and 1.21 and 6.53 mg mg_cat_^−1^ h^−1^ for 2D-Ni-N_4_-COF as the potential was varied from −0.5 V to −0.7 V *vs.* RHE. 3D-Ni-N_4_-COF exhibited NH_3_ productive rates that are 2.3−, 1.6−, and 1.4− times higher than that of 2D-Ni-N_4_-COF at −0.5, −0.6, and −0.7 V *vs.* RHE, respectively.

We also tested the durability of the 2D and 3D Ni–N_4_-COFs during nitrate reduction over eight consecutive electrolysis cycles in an H-cell reactor at −0.6 V *vs.* RHE for selective NH_3_ production (Fig. S17–19); the FEs and corresponding NH_3_ productivities are displayed in Fig. 4e, f, and S18. These COFs maintained FEs of above 80%, with activities increasing to varying degrees. Moreover, we also evaluated the chemical stability of 3D COF; it also demonstrated good durability in different solvents (Fig. S20). We deduced that the performance enhancement of NO_3_RR is not attributed to the changes of frameworks but to the local structure of catalytic sites. This observation may be related to the nitrate-reduction mechanism; consequently, we employed *in situ* spectroscopy to investigate this further.

**Fig. 5:**
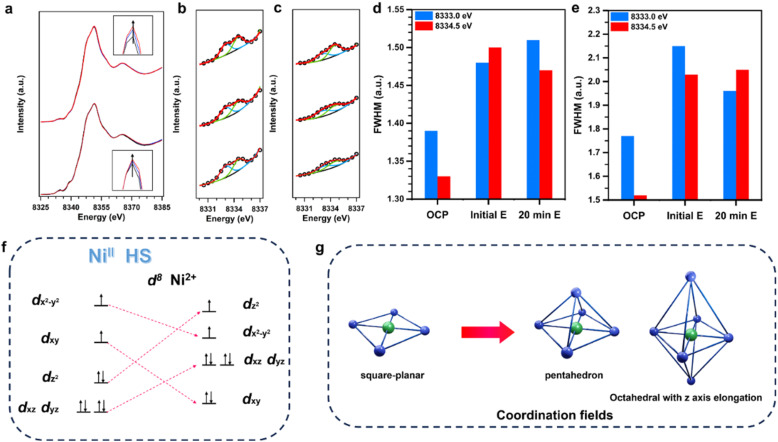
*In situ* X-ray absorption spectroscopy. (a) Normalized *operando* Ni K-edge XANES spectra for 2D Ni–N_4_–COF (up) and 3D Ni–N_4_–COF (down) various states (OCP, 0.6 V initial and after 20 min, for black, blue, and red curves, respectively) in 1 M KOH/0.5 M KNO_3_ aqueous solution at room temperature in Ar. The inset shows the enlarged Ni K-edge XANES spectra, up for 2D Ni–N_4_–COF and down for 3D Ni–N_4_–COF, respectively. The Gaussian fitting of XANES pre-edge peaks with different states from the (b) 2D Ni–N_4_–COF and (c) 3D Ni–N_4_–COF: up for OCP, middle for 0.6 V initial and down for 0.6 V after 20 min. The full width at half maxima (FWHM) of pre-edge fitting peaks from the (d) 2D Ni–N_4_–COF and (e) 3D Ni–N_4_–COF, respectively. (f) The deduced 3d electron configuration of Ni centers in two COFs. (g) The corresponding coordination fields transformation of the Ni centers in two COFs.

*In situ* XAFS was used to investigate the electronic states and coordination environments of the Ni sites during the nitrate-reduction reaction. EXAFS spectra were acquired at the open-circuit potential (OCP), 0.6 V, and after reacting for 20 min at 0.6 V. The XANES profiles of the three states are fundamentally the same, which implies that the average coordination environments of the Ni atoms are not significantly different (Fig. 5a). We peak-fitted the wide pre-edge peaks to clarify the Ni electronic states, which revealed overlapping peaks at 8333.0 and 8334.5 eV (Fig. 5b) that were approximately equal in area, which implies that 2D-Ni-N_4_-COF and 3D-Ni-N_4_-COF contain two high-spin, high-energy 3d-orbital holes (Fig. 5d–f). We identified quadrupole 1s to 3d_*xy*_ and 3d_*x*^2^−*y*^2^_ transitions. However, we also observed that the full width at half maximum of each fitted peak increased as the magnitude of the working potential increased, which we ascribe to the extended energy levels associated with the 3d orbitals because the areal peak ratio remained constant (Fig. 5f). This observation is consistent with the configuration evolving from Ni–N_4_ to five- or six-coordination (Fig. 5g). The five-coordinated tetragonal pyramidal configuration and Jahn–Teller compression of the *z*-axis of the six-coordinated octahedron result in similar 3d orbital splitting; therefore, the co-existence of four-, five-, and six-coordination leads to the observed energy-level broadening.

**Fig. 6:**
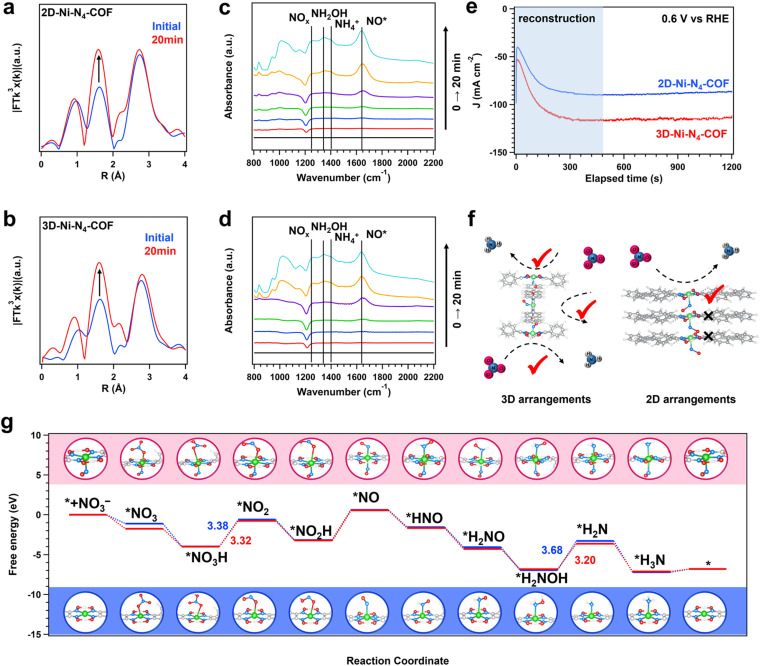
Mechanism studies. Fourier transform magnitudes of EXAFS spectra (without phase correction) of 2D Ni–N_4_-COF (a) and 3D Ni–N_4_-COF (b) at 0.6 V with different time stages (initial and after 20 min, for blue and red curves, respectively) in 1 M KOH/0.5 M KNO_3_ aqueous solution at room temperature in Ar. *In situ* time-resolved ATR-FTIR spectroscopy measurements under 0.6 V *vs.* RHE for 2D Ni–N_4_-COF (c) and 3D Ni–N_4_-COF (d) during electrocatalytic reduction of nitrate. (e) Chronoamperometric (*i*–*t*) curve of 2D Ni–N_4_-COF (a) and 3D Ni–N_4_-COF (b) at 0.6 V in 1 M KOH/0.5 M KNO_3_ aqueous solution at room temperature in Ar. (f) The deduced mechanism of different arrangements of Ni–N_4_ sites for activity. (g) Gibbs free energy diagram *via* the minimum energy pathway and corresponding adsorption configurations of various intermediates generated during NO_3_RR.

FT *k*^3^-weighted EXAFS spectra were also acquired for the 2D and 3D Ni–N_4_-COFs to further verify the abovementioned gradual configurational evolution; the results for various reaction stages are shown in Fig. 6a and b. The intensity of the peak at around 1.55 Å was observed to increase during nitrate reduction over 20 min, owing to increased first-shell coordination through the axial adsorption of species by the nickel glyoximate units. Moreover, we also observed shrinkage of the nickel centers in the first shells of both COFs, which implies that axial adsorption involves a shorter bond than the glyoximate Ni–N bond, while also demonstrating elongation of the *z*-axis of the six-coordinated octahedron. We also used *in situ* attenuated total reflectance Fourier-transform infrared (ATR-FTIR) spectroscopy to acquire molecular-level information by observing the vibrations of the intermediates involved in nitrate reduction (Fig. 6c and d). 2D-Ni-N_4_-COF exhibited peaks at 1641, 1409, 1347, and 1266 cm^−1^ that are attributable to *NO, NH_4_^+^, NH_2_OH, and NO_*x*_ species, respectively, while 3D-Ni-N_4_-COF exhibited the same species under the same conditions, with peaks observed at 1639, 1407, 1350, and 1260 cm^−1^. *NO was first observed in the time-resolved spectra, while other reaction species were not. Considering that NO_*X*_ is a reduced species formed prior to *NO, their peaks are also much less intense than that corresponding to *NO, which is ascribable to nitrate reduction to *NO and stagnation on the Ni sites. Taken together, the ATR-FTIR and *in situ* XAS results reveal that *NO are the axially adsorbed species at the nickel glyoximate unit. The coordination number change reflected the reconstruction degree of Ni–N_4_ in two COFs, the estimated reconstruction proportion in 2D COFs of ∼40% and 3D COFs of ∼100% (Fig. S21, 22, Tables S8 and S9).

The chronoamperometry curves were observed to significantly intensify with time due to the formation of Ni–N_4_–NO. The 2D and 3D Ni–N_4_-COFs exhibited currents that significantly increased over several minutes, consistent with slow changes in activity. Moreover, while the 2D and 3D COFs exhibited similar initial currents, their maximum currents differed significantly during the reaction. These observations are consistent with the slow reconstruction of each COF to the corresponding five-coordinated state and that 3D-Ni-N_4_-COF is more active following reconstruction than 2D-Ni-N_4_-COF. The 2D and 3D COFs appear to be arranged differently, despite containing the same Ni sites. The 2D COF layers are AA-stacked with inter-layer distances of about 3 Å; the Ni sites that participate further in the NO_3_RR following reconstruction need more space but are limited by this stacking arrangement (Fig. 6f). In contrast, the isolated Ni sites in the 3D arrangement avoid such steric hindrance and are thus more accessible and highly active.

To reveal the active rules of the reconstruction sites and initial sites at an atomic level, the density functional theory (DFT) calculation was employed (Fig. 6g, Tables S10 and S11). Combined with *in situ* spectra data, the NO_3_RR pathway on the Ni–N_4_ and Ni–N_4_–NO sites followed a typical 8-electron process, and the intermediates *NO_3_, *NO_3_H, *NO, *NO_2_, *NO_2_H, *NO, *HNO, *H_2_NO, *H_2_NOH, *H_2_N, and *H_3_N were obtained in the catalytic process. The formation of *NO_3_ was spontaneous adsorption, and the free energy change (Δ*G*) on Ni–N_4_–NO was −1.75 eV, smaller than that of the Ni–N_4_ (−1.12 eV), suggesting the stronger binding ability of *NO_3_. Subsequently, the three energy barriers led to the activity differences of Ni–N_4_ and Ni–N_4_–NO sites, which were the steps for *NO_3_H to *NO, *NO_2_H to *NO, and *H_2_NOH to *H_2_N. The calculated Δ*G* for Ni–N_4_ were 3.38, 3.79, and 3.68 eV, while Ni–N_4_–NO had smaller Δ*G* for the same steps: 3.32, 3.78, and 3.20 eV, respectively. At the final step to release the adsorbed *NH_3_, the reconstructed sites also exhibited an advantage since the 0.1 eV energy barrier was lower than that of the initial site. Thus, we have determined that the reconstructed sites of Ni–N_4_–NO exhibit more activity than that of the initial Ni–N_4_ sites for NO_3_RR to NH_3_.

## Conclusions

In summary, well-designed 2D or 3D-arranged catalytic COF sites are highly active and selective for NH_3_ electrosynthesis in a wide potential range. 3D-Ni-N_4_-COF exhibited NH_3_ production rates that are 2.3, 1.6, and 1.4 times higher than those of 2D-Ni-N_4_-COF at −0.5, −0.6, and −0.7 V *vs.* RHE, respectively. *In situ* XAFS and ATR-FTIR experiments revealed that Ni–N_4_ sites become reconstructed into more active Ni–N_4_-NO sites as the reaction progresses. The 3D COF undergoes reconstruction more easily and offers more accessible reaction sites on account of its higher degree of spatial freedom. This work not only revealed a structure–activity relationship for catalytically active sites but also provided dimensional insight for the design of active-center architectures.

## Author contributions

Conceptualization: S. Y., Q. X., L. Z.; methodology: S. Y., S. B,; investigation: S. Y., S. B., Q. X.; visualization: S. Y., Q. X.; funding acquisition: L. Z., S. B., Q. X.; project administration: Q. X., L. Z.; supervision: S. B., L. Z., Q. X.; writing—original draft: S. Y., Q. X.; writing—review and editing: S. Y., S. B., L. Z., Q. X.

## Conflicts of interest

There are no conflicts to declare.

## Supplementary Material

SC-OLF-D5SC02151K-s001

## Data Availability

All the data supporting this article have been included in the main text and the ESI. The data supporting this article have been included as part of the ESI. See DOI: https://doi.org/10.1039/d5sc02151k.
