# Supplementary material for: Modulating the direction of catalytic glyoximate sites of covalent organic frameworks towards electrocatalytic nitrate reduction
Source: Chem Sci. 2025 Jul 21. Online ahead of print. doi: 10.1039/d5sc02151k (PMC12315718; doi:10.1039/d5sc02151k)
Supplement: Supplementary file 1 [file SC-OLF-D5SC02151K-s001.pdf]

Supplementary Information (SI) for Chemical Science.
This journal is © The Royal Society of Chemistry 2025

Supporting Information

Modulating the Direction of Catalytic Glyoxime Sites of Covalent Organic Frameworks towards Electrocatalytic Nitrate Reduction

S. Yang et al.

Experimental Procedures

Materials. 4,4',4'',4'''-(1,4-phenylenebis(azanetriyl))tetrabenzaldehyde, 4,4',4''-nitrilotribenzaldehyde were purchased from Jilin Chinese Academy of Sciences-Yanshen Technology Co., Ltd. KOH (90%, Greagent), KNO_3 (99.0%, Sinopharm Chemical Reagent Co., Ltd), 0.1 M NaClO solution (Shanghai Macklin Biochemical Co., Ltd), deionized water (18 MΩ), $C_5FeN_6Na_2O \cdot 2H_2O$ (99.0%, Shanghai Macklin Biochemical Co., Ltd), salicylic acid (99.0%, Shanghai Aladdin Biochemical Technology Co., Ltd.) and Trisodium citrate dihydrate (99.5%, Sinopharm Chemical Reagent Co., Ltd) used as received without further purification. Nickel coordinated glyoximate di-biphenyl amine (Ni-DBA) was synthesized according to the previous report [1].

Synthesis of 2D-Ni-N_4-COF. Nickel coordinated glyoximate di-biphenyl amine (Ni-DBA) (18 mg, 0.02 mmol) and N,N,N',N'-Tetrakis(4-aminophenyl)-1,4-benzenediamine (10.49 mg, 0.02 mmol) were placed in a 10 ml ampoules with o-DCB (0.6 mL), *i*-PrOH (0.4 mL), and acetic acid (6.0 M, 0.2 mL), respectively [1]. After ultrasonic dispersion for 5 minutes, freeze and vacuum with liquid nitrogen before thawing. Repeat this process three times and seal with a flame. Then the tube was heat in a 120 °C oven for 5 days to finish the condensation reaction. After cooling to room temperature, the products were centrifuged and washed several times with DMF, THF, and ethanol. Finally, the resulting solid was vacuum dried at 80 °C for 12 hours to obtain the yellow solid (81% yield).

Synthesis of 3D-Ni-N_4-COF. Nickel coordinated glyoximate di-biphenyl amine (Ni-DBA) (18 mg, 0.02 mmol) and tris(4-aminophenyl)amine (8.78, 0.027 mmol) were placed in a 10 ml ampoules with o-DCB (0.6 mL), *i*-PrOH (0.4 mL), and acetic acid (6.0 M, 0.2 mL), respectively [1]. After ultrasonic dispersion for 5 minutes, freeze and vacuum with liquid nitrogen before thawing. Repeat this process three times and seal with a flame. Then the tube was heat in a 120 °C oven for 5 days to finish the condensation reaction. After cooling to room temperature, the products were centrifuged and washed several times with DMF, THF, and ethanol. Finally, the resulting solid was vacuum dried at 80 °C for 12 hours to obtain the yellow solid (78% yield).

Physical characterization. Powder X-ray diffraction (PXRD) data were recorded on an Ultima IV diffractometer with CuK_α a radiation by depositing powder on a glass substrate, from $2\theta = 3$ to 30° with increments of 0.02°. Nitrogen sorption isotherms were measured at 77 K with a TriStar II instrument (Micromeritics). X-ray photoelectron spectroscopy (XPS) experiments were performed on a Thermo Scientific K-Alpha XPS spectrometer using an AlK_α X-ray radiation source. The Brunauer–Emmett–Teller (BET) method was utilized to calculate the specific surface areas. By using the non-local density functional theory (NLDFT) model, the pore volume was derived from the sorption curve. HRTEM images were obtained with a transmission electron microscope (TEM, FEI Tecnai G2). FE-SEM images were obtained on a FEI Sirion-200 or Hitachi high technologies (SU-6600) field-emission scanning electron microscope at an electric voltage of 5 KV. EDX and elemental mapping were acquired using a HITCHI Miniscope TM3030.

Electrochemical NO_3^-RR measurements. Before tests, the Nafion 117 membrane was pretreated by heating it in H_2O_2 (5%) aqueous solution at 80 °C for 1 h and ultrapure water at 80 °C for another

1 h, respectively, followed by treatment in 0.05 M H_2SO_4 for 1 h and ultrapure water for another 3 h. The electrochemical experiments were conducted on a CHI 760e electrochemical analyser by using a three-electrode configuration with an H-Cell (working electrode, counter electrode of Pt gauze and reference electrode of Ag/AgCl/saturated KCl). All potentials were converted to RHE. For electrochemical NO_3^-RR tests, potentiostatic tests were carried out in 1 M KOH / 0.5 M KNO_3 with feeding Ar. The volume of the electrolyte in the anode and cathode chamber is 25 ml for each. The electrolyte in the cathodic compartment was stirred during electrolysis.

Typically, approximately 5 mg catalyst, 5 mg carbon black and 20 μl Nafion solution (5 wt.%) were dispersed in 480 μl absolute ethanol by sonicating for 2 h to form a homogeneous ink. Then 100 μl of homogeneous ink was loaded onto a hydrophilic carbon paper electrode (Suzhou Sinero technology Co.) with an area of 1×1 cm^2 with a graphitic electrode holder. The average mass loading is ~1 mg_{cata} cm^{-2}. The applied potentials were all converted to the RHE scale using the following relation:

$$E \text{ (versus RHE)} = E \text{ (versus Ag/AgCl)} + 0.197 \text{ V} + 0.059 \text{ V} \times \text{PH} \quad (1)$$

Determination of ammonia. As-produced ammonia was spectrophotometrically determined by the indophenol blue method with modification after diluting the post-test electrolytes to appropriate concentration to match the range of calibration curves. First, a 1.3 ml of the solution (or diluted for UV-Vis test range) was taken from the electrochemical reaction vessel. Then 2 ml of a 1 M NaOH solution containing 5 wt.% salicylic acid and 5 wt.% sodium citrate was added, followed by addition of 0.5 ml of 0.1 M NaClO and 0.2 ml of an aqueous solution of 1 wt.% $C_5FeN_6Na_2O$ (sodium nitroferricyanide). After 2 h at room temperature in darkness, the absorption spectrum was measured using a UV–Vis spectrophotometer (MAPADA UV-1800PC). The formation of indophenol blue was determined using the absorbance at a wavelength of 654 nm. The concentration–absorbance curves were calibrated using standard ammonia sulfate solutions, as shown in Fig. S14, which contained the same concentrations of electrolytes as used in the electrocatalysis experiments.

In situ XAS measurements. The in situ XAS spectra at the Ni K-edge were recorded at the BL14W1 beamline of Shanghai Synchrotron Radiation Facility [2]. The electron storage ring operated at 3.5 GeV. The beam current of the storage ring was 220 mA in a top-up mode. The incident photons were monochromatized by a Si (111) double-crystal monochromator. The energy calibration was performed using a Ni foil. The in situ XAS spectra were recorded in the fluorescence mode using a Lytle detector. The electrolysis was performed in Ar-saturated 1 M KOH /0.5 M KNO_3 solution in a two-compartment H-shape cell. The sample was at 45° with respect to the incident beam direction. The electrodes were prepared as described in the"Electrochemical NO_3^-RR measurements." section. Ar was bubbled into the cell with a constant flow rate during the *in situ* experiments. Kapton tape was used to seal the cell. The operando in situ spectra were measured at a potential -0.6 V vs RHE (keeping a constant potential when recording the spectrum). XAS data were analyzed using the Athena software [3].

XAFS data processing. Utilizing the ATHENA module of the IFEFFIT software packages, the obtained EXAFS data were performed according to the standard procedures[3]. The EXAFS contributions were separated from different coordination shells by using a hanning windows.

Subsequently, the quantitative curve-fittings were carried out in the R-space with a Fourier transform k-space range using the module ARTEMIS of IFEFFIT. During the curve-fitting, the overall amplitude reduction factor S_0^2 was fixed to the best-fit value determined from fitting the data of metal foil. For the sample, the structural parameters, such as the coordination number N, interatomic distance R, the Debye-Waller factor σ^2 and the edge-energy shift ΔE_0 were allowed to vary during the fitting process.

Calculations. All calculations were carried out based on density functional theory (DFT) as implemented in Vienna ab initio simulation package (VASP) with exchange-correlation functional of generalized gradient approximation (GGA) of Perdew, Burke, and Ernzerhof (PBE) method. A grid of 1 × 1 × 1 Monkhorst–Pack k-points was used for the structural relaxation. A vacuum layer of 15 Å is adopted in the direction perpendicular to the monolayer surface to avoid the interactions between periodic slabs. The energy cutoff was set to be 450 eV. The convergence criterion for the energy and maximum force for the optimization were set to 10^{-5} eV and 0.05 eV/Å, respectively. To further explain the relative reaction trend, the free energy changes are calculated according to the following equation,

$$\Delta G = \Delta E + \Delta E_{ZPE} - T\Delta S$$

where ΔE, ΔE_{ZPE}, and ΔS are the differences of total energy, zero point energy, and entropy between the product and reactants, respectively. Here, we just consider the ground states of containing species at temperature of 0 K. So, the contribution from the entropy term is $298.15\times\Delta S$.

Results and Discussion

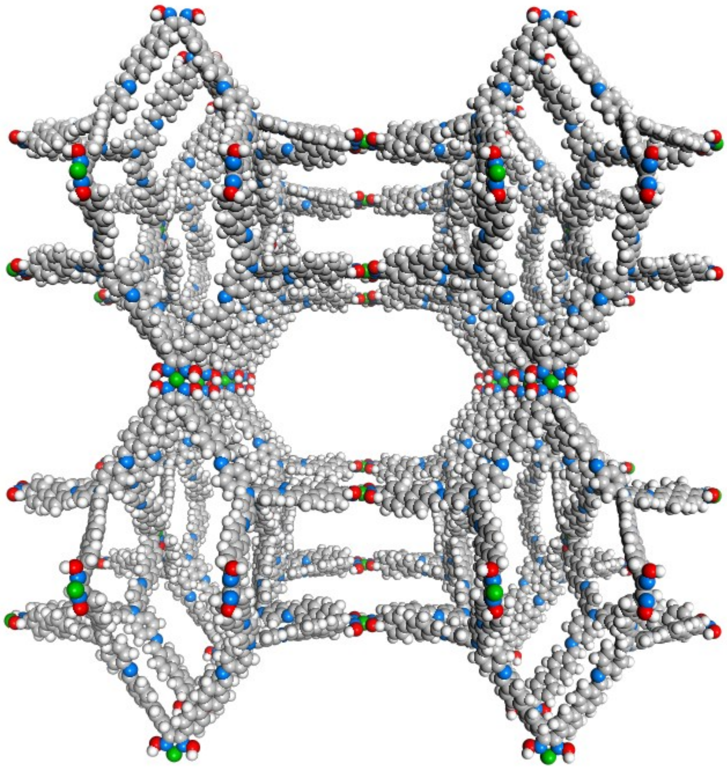

Fig. S1. Side view of pto topological 3D-Ni-N_4-COF.

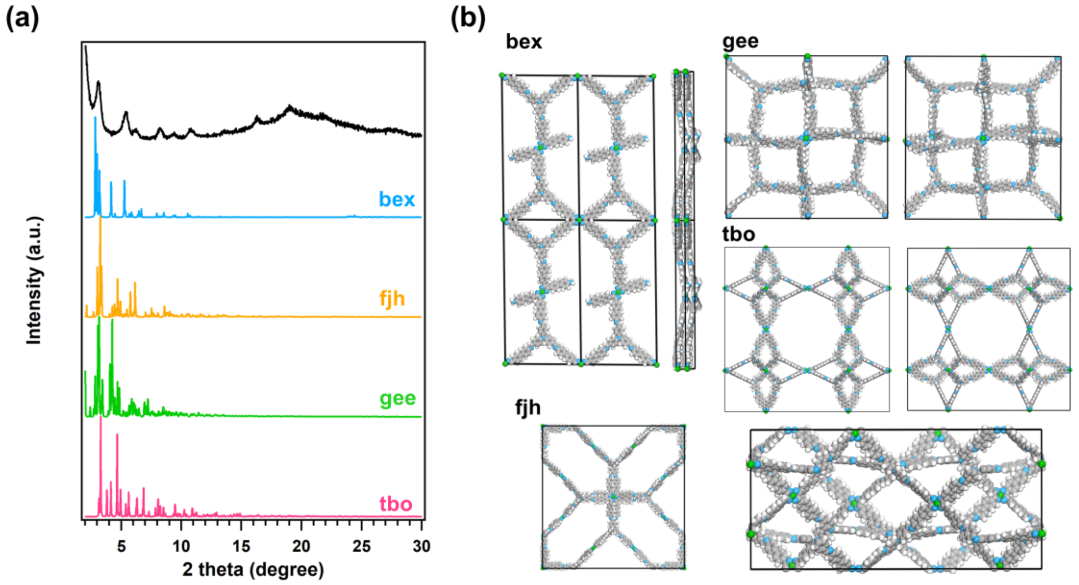

Fig. S2. (a) The experimental PXRD pattern (black curve) of 3D-Ni-COF compared to the simulation XRD curves of bex (blue curve), fjh (yellow curve), gee (green curve), and tbo (pink curve) topology's crystal models. (b) Corresponding structural diagram.

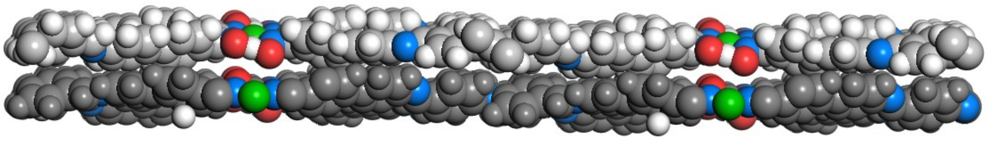

Fig. S3. Side view of AB-stacking 2D-Ni-N_4-COF.

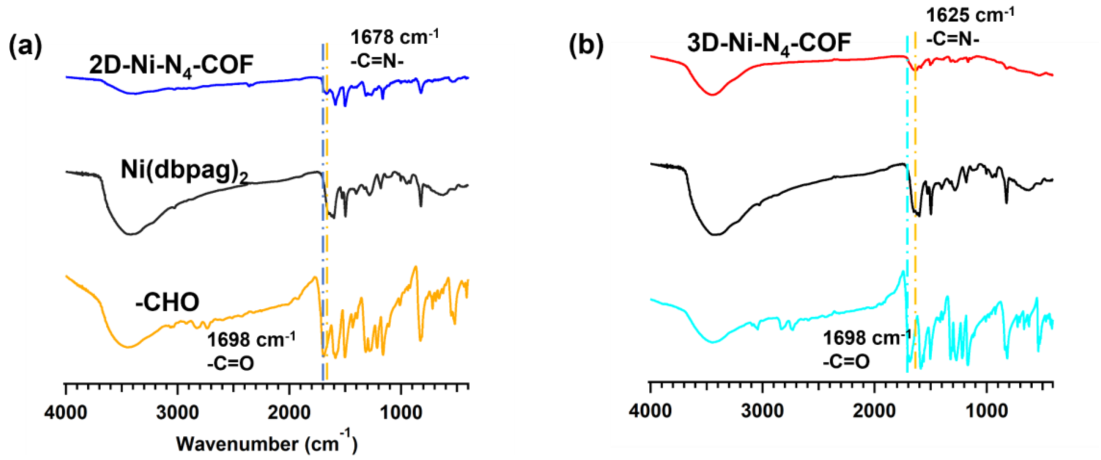

Fig. S4. FT-IR of 2D-Ni-N_4-COF (blue curve), 3D-Ni-N_4-COF (red curve), and their building units (N,N,N',N'-Tetrakis(4-aminophenyl)-1,4-benzenediamine for yellow curve, Ni(dbpag)$_2$ for black curve and tris(4-aminophenyl)amine for blue curve).

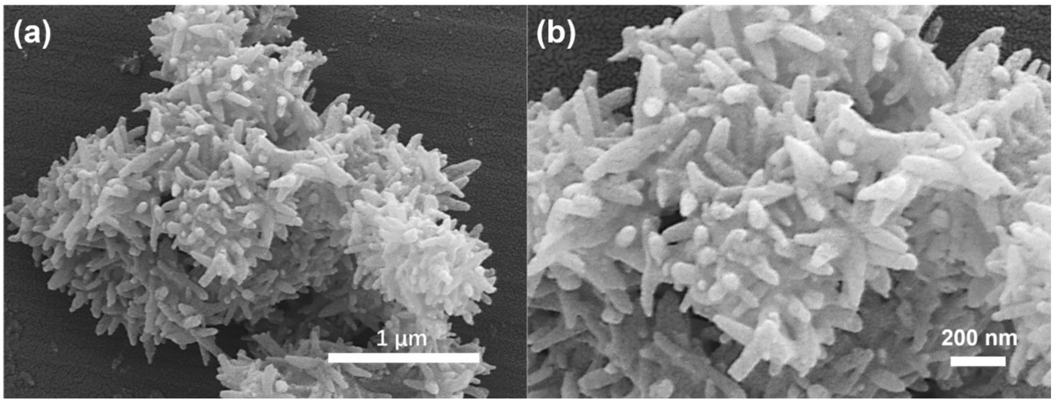

Fig. S5. SEM images of 2D-Ni-N_4-COF.

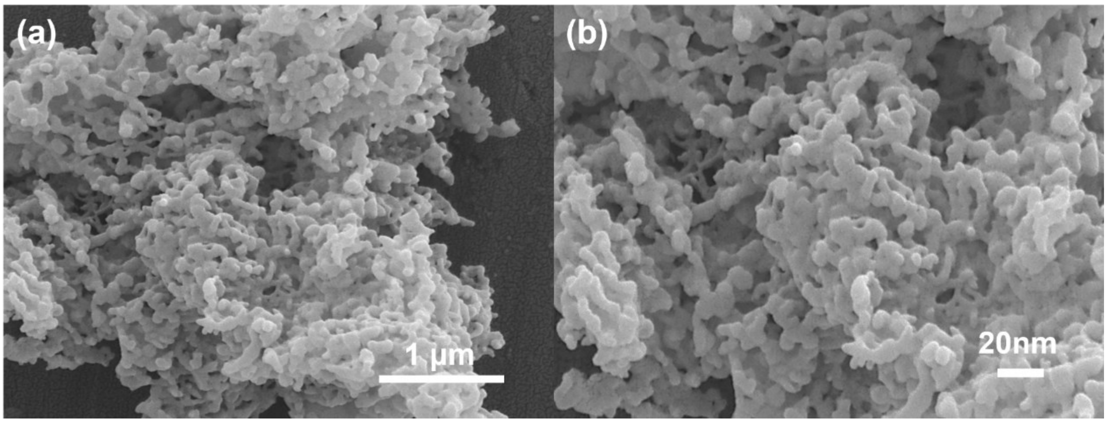

Fig. S6. SEM images of 3D-Ni-N_4-COF.

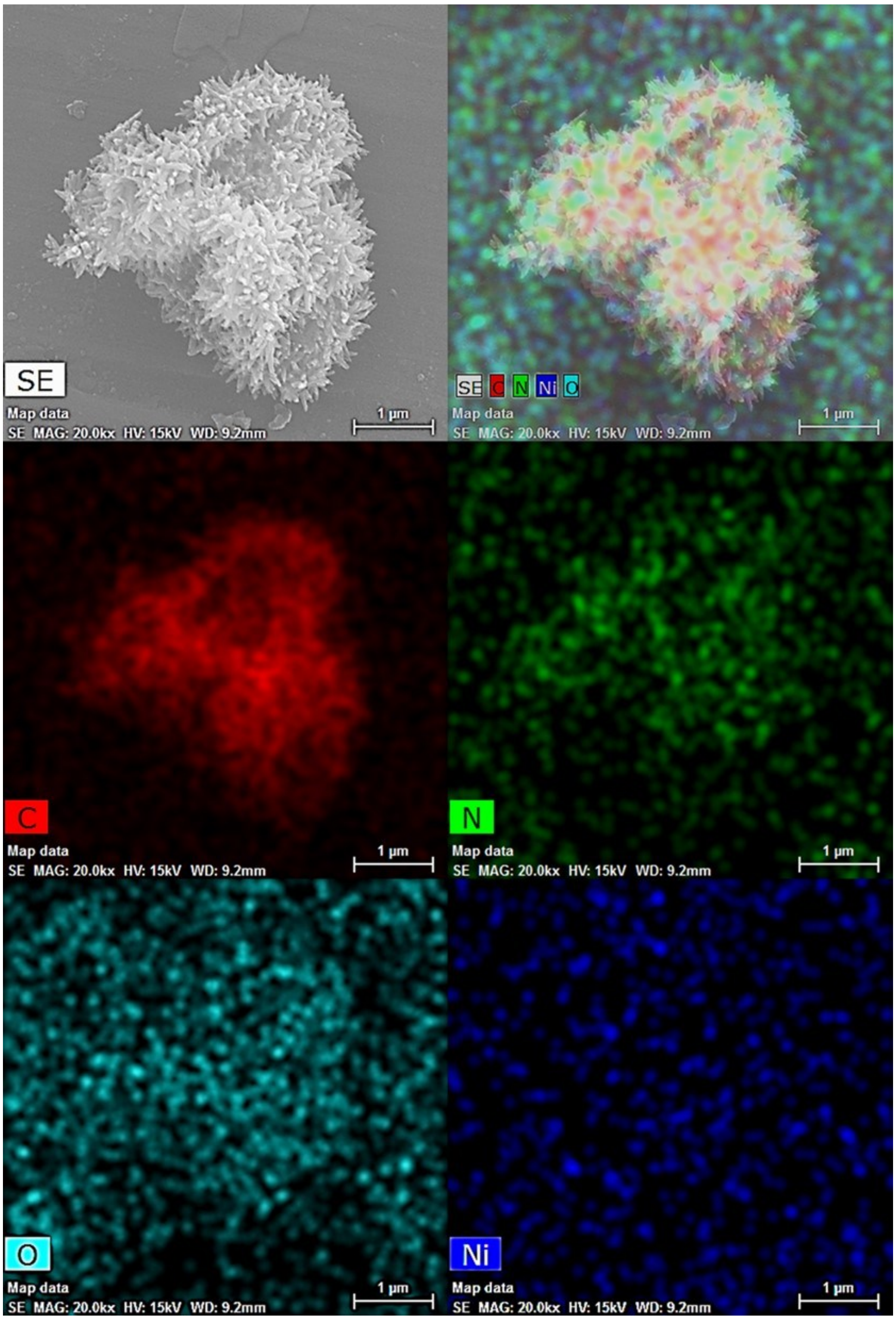

Fig. S7. EDS mapping of 2D-Ni-N_4-COF reveals the homogeneous distribution of C, N, O, and Ni elements.

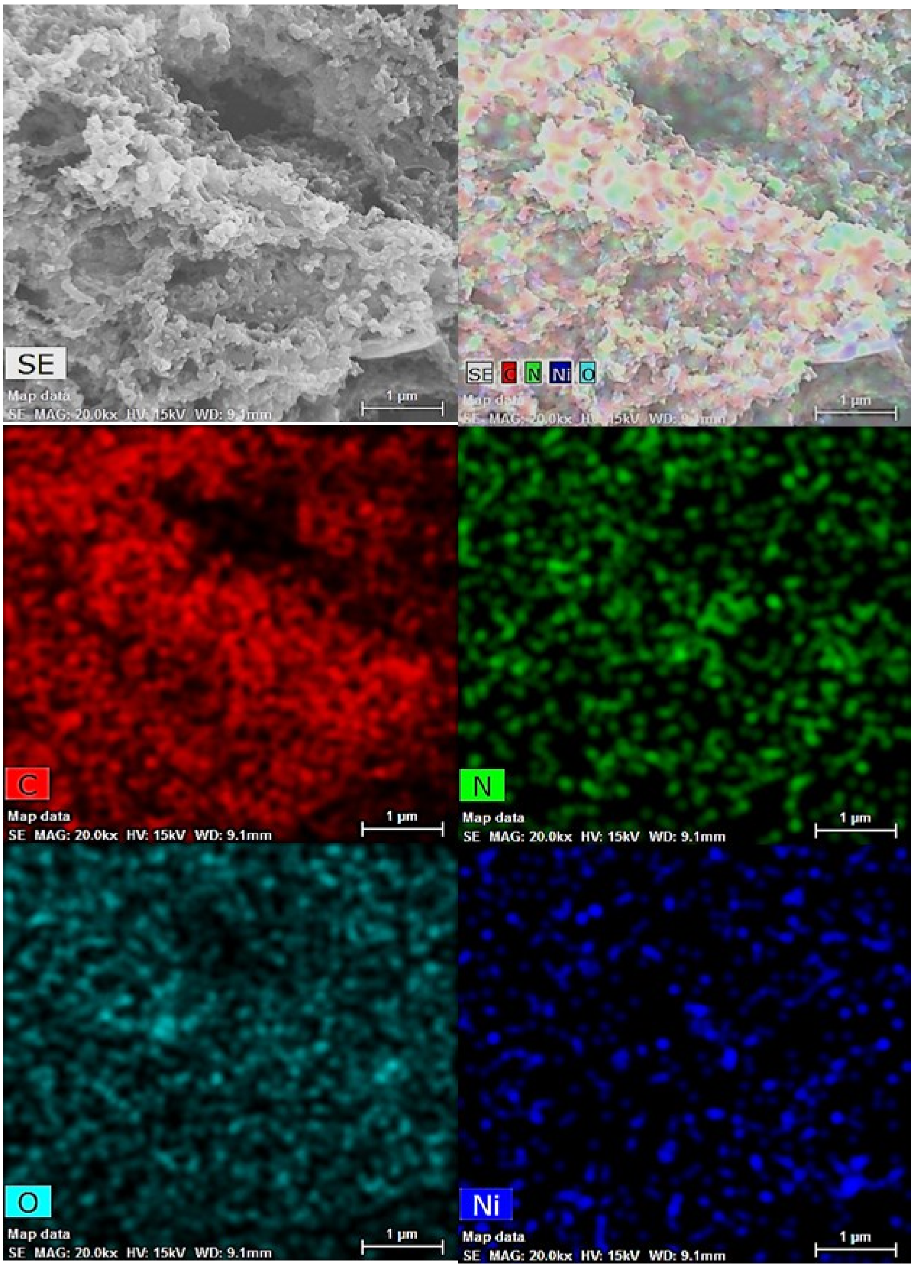

Fig. S8. EDS mapping of 3D-Ni-N_4-COF reveals the homogeneous distribution of C, N, O, and Ni elements.

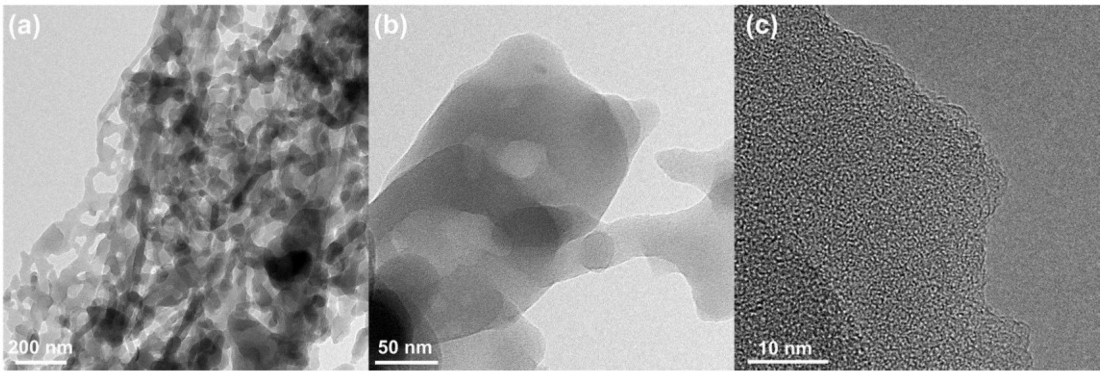

Fig. S9. (a, b) TEM and (c) HR-TEM images of 2D-Ni-N_4-COF.

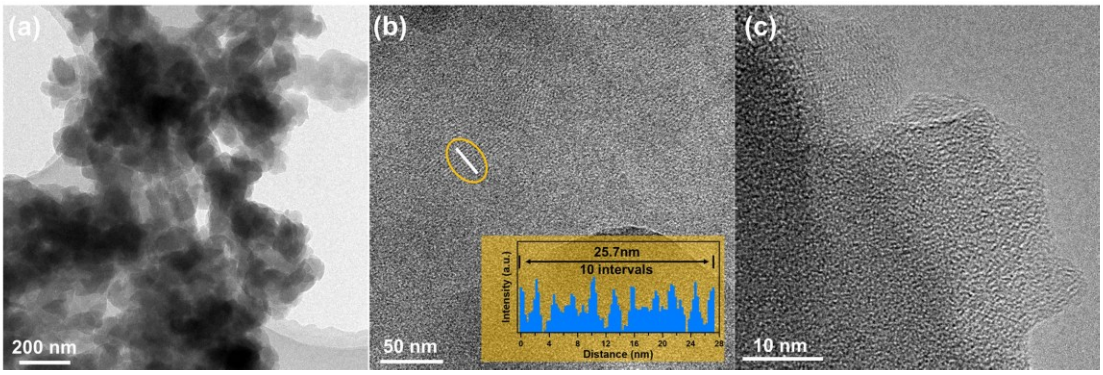

Fig. S10. (a, b) TEM and (c) HR-TEM images of 3D-Ni-N_4-COF.

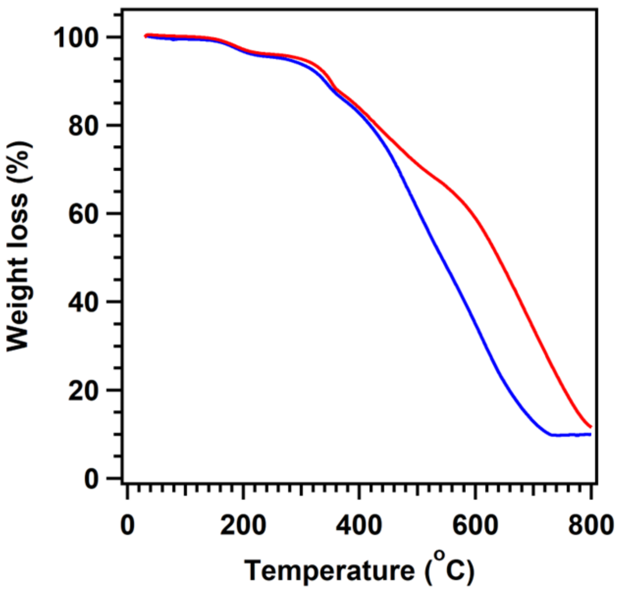

Fig. S11. TGA profiles of 2D-Ni-N_4-COF (blue curve) and 3D-Ni-N_4-COF (red curve) from room temperature to 800 °C under N_2.

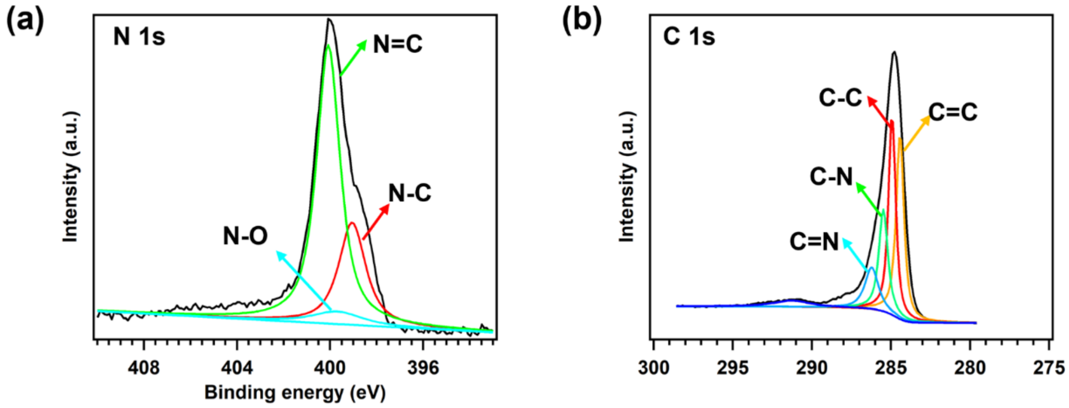

Fig. S12. High resolution XPS spectra of the 2D-Ni-N_4-COF: (a) N 1s spectrum and (b) C 1s spectrum.

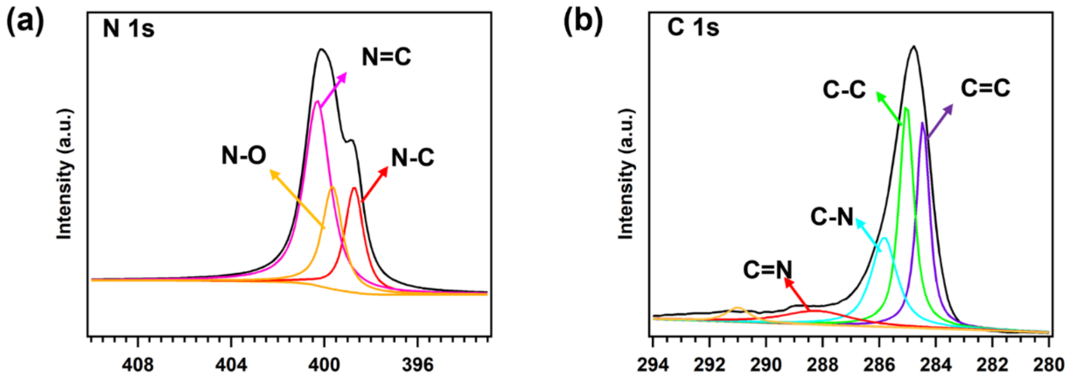

Fig. S13. High resolution XPS spectra of the 3D-Ni-N_4-COF: (a) N 1s spectrum and (b) C 1s spectrum.

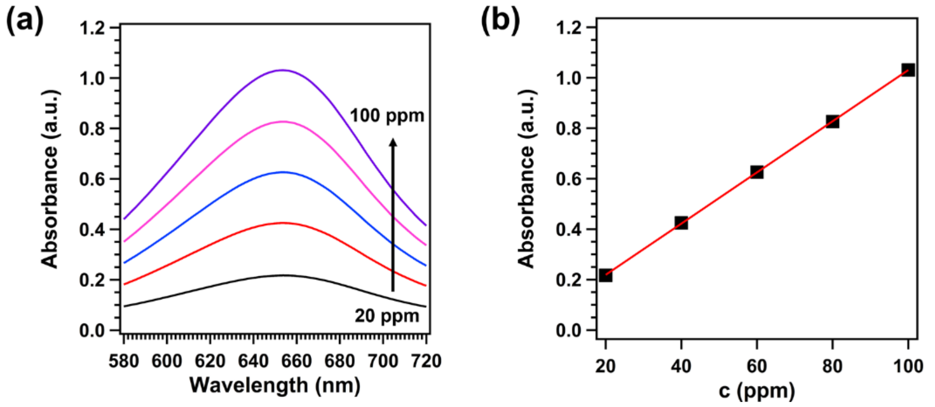

Fig. S14. Absolute calibration of the indophenol blue method using ammonium chloride solutions of known concentration as standards. (a) UV-Vis curves of indophenol assays with NH^{4+} ions after incubated for 2 hours, the NH^{4+} concertation corresponds to the solutions in cuvettes. (b) Calibration curve used for estimation of NH3 by NH^{4+} ion concentration. The absorbance at 654 nm was measured by UV-Vis spectrophotometer, and the fitting curve shows good linear relation of absorbance with NH^{4+} ion concentration of three times independent calibration curves.

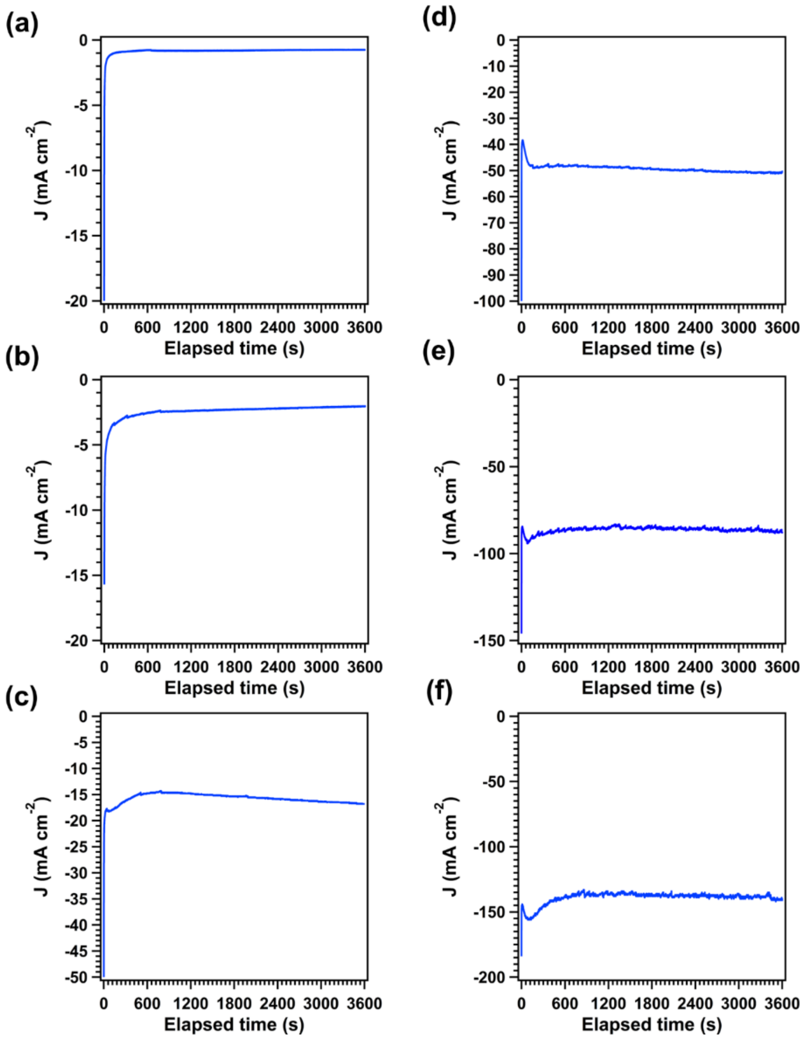

Fig. S15. Chronoamperometry results of 2D-Ni-N_4-COF at the potential of (a) 0.3 V, (b) 0.4 V, (c) 0.5 V, (d) 0.6 V, (e) 0.7 V, and (f) 0.8 V, respectively.

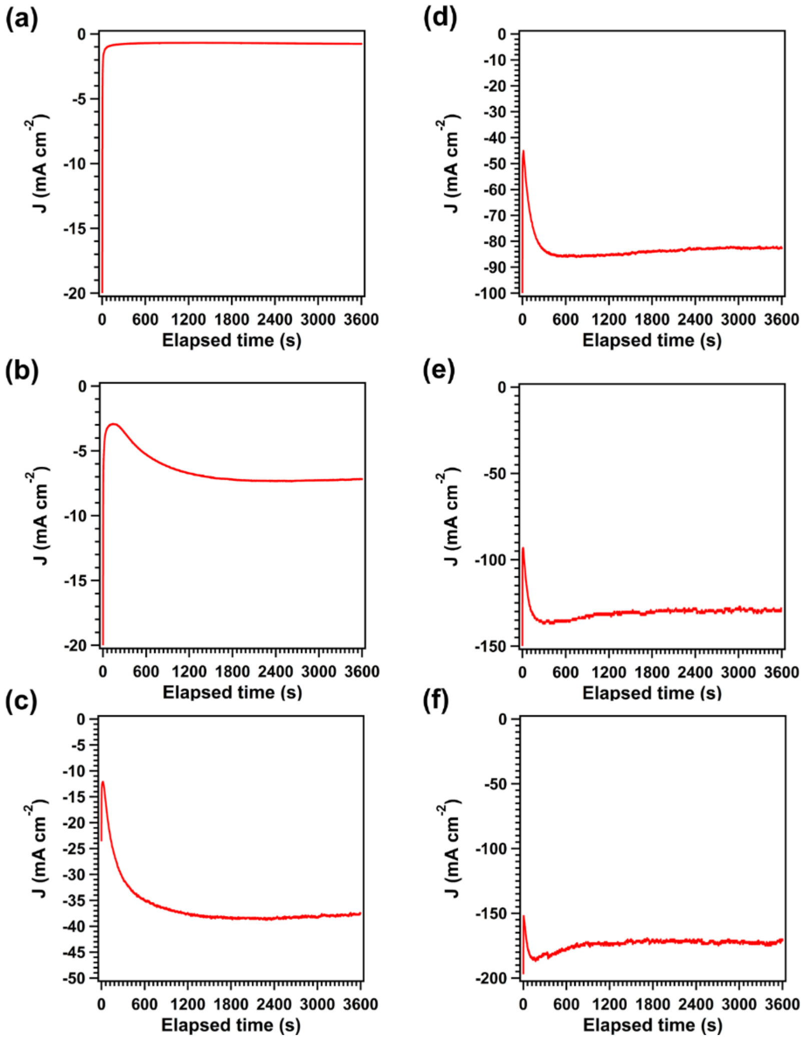

Fig. S16. Chronoamperometry results of 3D-Ni-N_4-COF at the potential of (a) 0.3 V, (b) 0.4 V, (c) 0.5 V, (d) 0.6 V, (e) 0.7 V, and (f) 0.8 V, respectively.

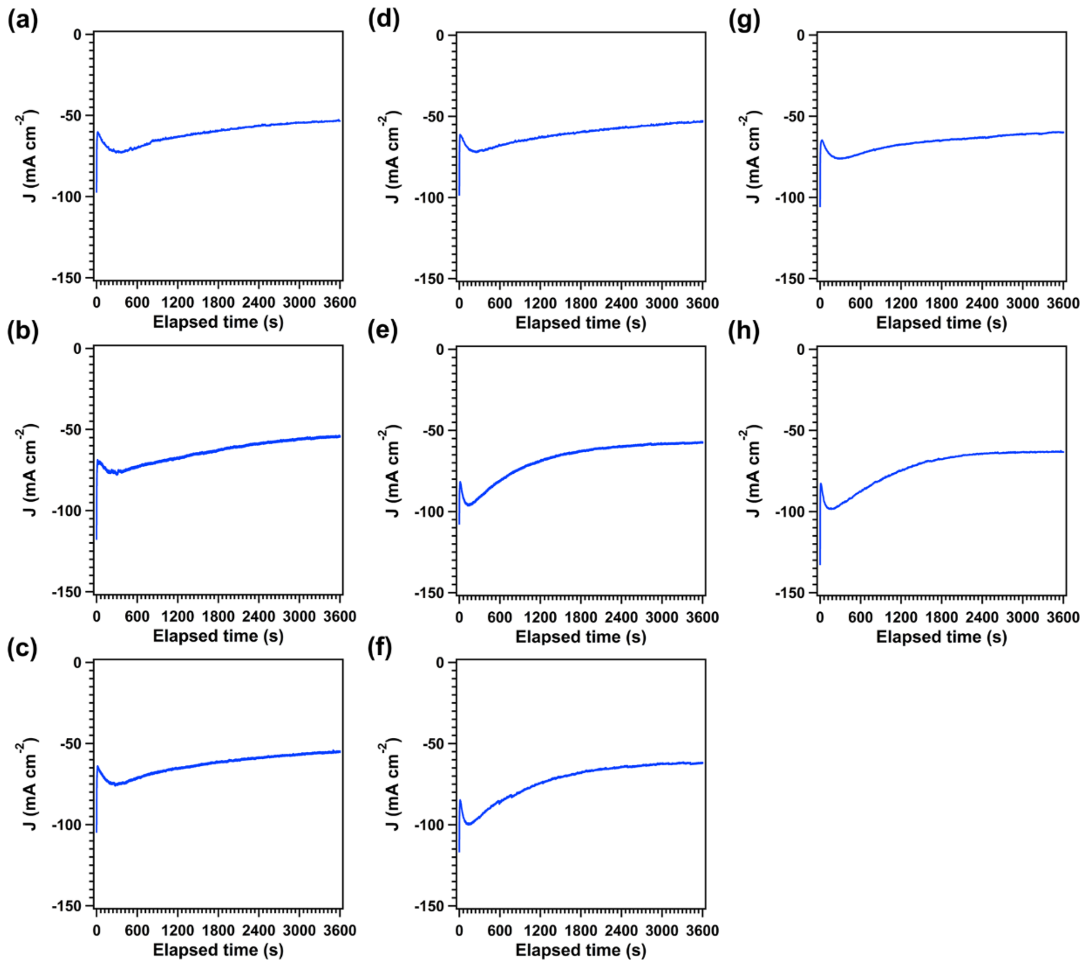

Fig. S17. Cycling test of 2D-Ni-N_4-COF at -0.6 V vs RHE. Chronoamperometry results of 2D-Ni-N_4-COF recorded from cycle 1 to cycle 8 (a-h).

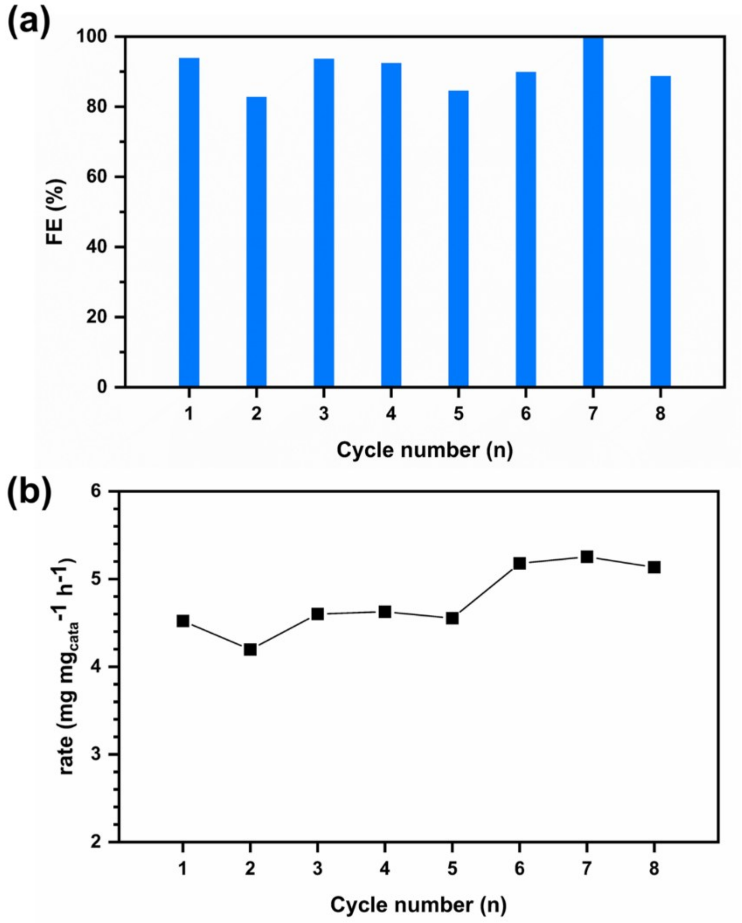

Fig. S18. The (a) average NH_3 faradaic efficiency and (b) production rate within each cycle for 2D-Ni-N_4-COF.

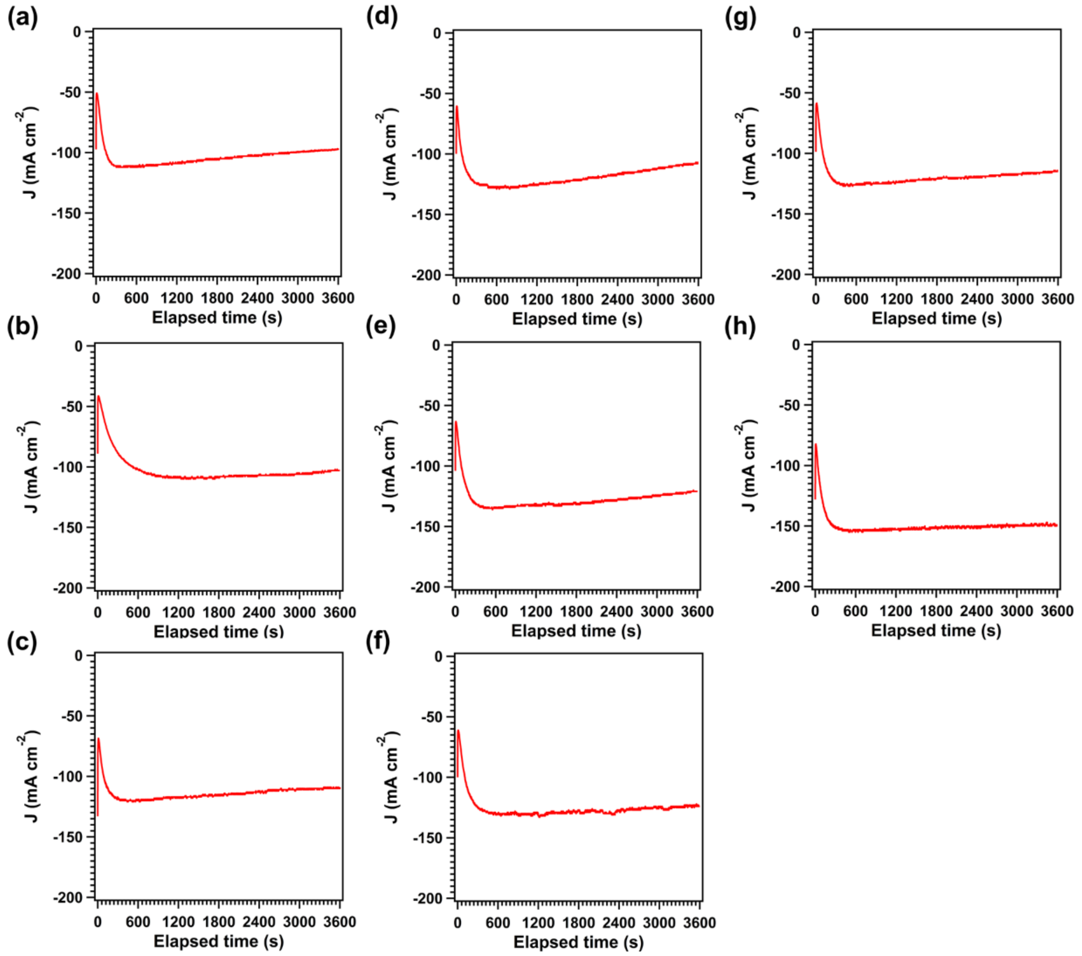

Fig. S19. Cycling test of 3D-Ni-N_4-COF at -0.6 V vs RHE. Chronoamperometry results of 3D-Ni-N_4-COF recorded from cycle 1 to cycle 8 (a-h).

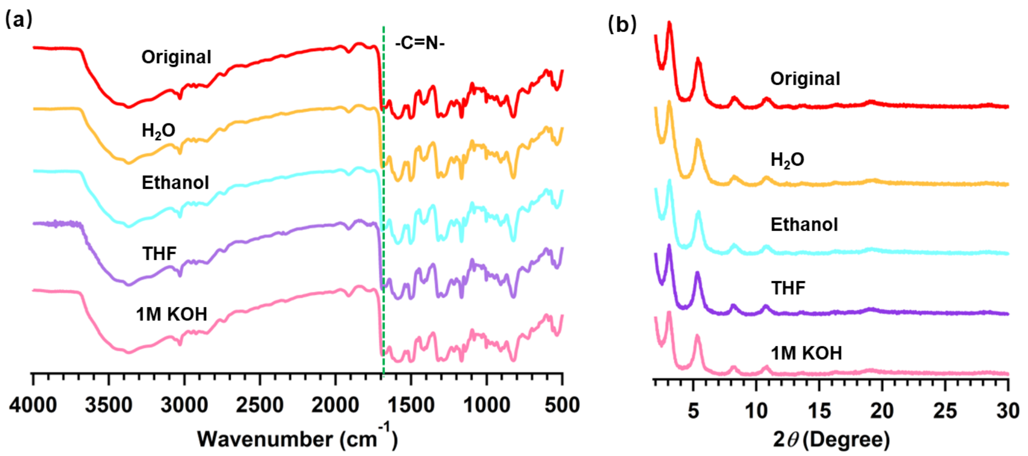

Fig. S20. FT-IR spectra (a) and PXRD pattern (b) of 3D-Ni-N_4-COF before and after chemical stability measurement.

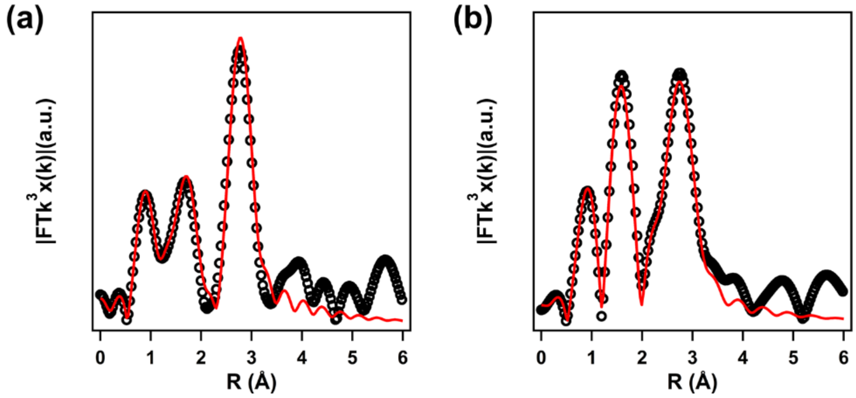

Fig. S21. Ni K-edge k^3-weighted Fourier transform spectra from EXAFS of 2D-Ni-N_4-COF at in situ tests, with the state of (a) 0.6 V, and (b) after reacting for 20 min at 0.6 V.

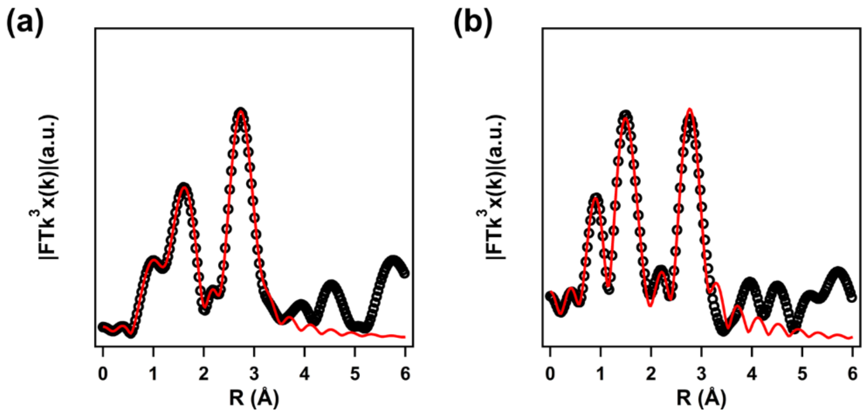

Fig. S22. Ni K-edge k^3-weighted Fourier transform spectra from EXAFS of 3D-Ni-N_4-COF at in situ tests, with the state of (a) 0.6 V, and (b) after reacting for 20 min at 0.6 V.

Table S1. Fractional atomistic coordinates for Pawley-refined unit cell parameters of 3D-Ni-N_4-COF with ffc topology. Space group CMCM, a = 15.3606 Å, b = 112.3527 Å, c = 32.8296 Å, $\alpha = \gamma = \beta = 90°$.

C1	C	0.63909	0.63068	0.32745	0	Uiso	1
C2	C	0.72661	0.69428	0.70434	0	Uiso	1
C3	C	0.7244	0.68809	0.66728	0	Uiso	1
C4	C	0.66321	0.69119	0.63743	0	Uiso	1
C5	C	0.60471	0.70041	0.64495	0	Uiso	1
C6	C	0.60678	0.70652	0.68186	0	Uiso	1
C7	C	0.66796	0.70363	0.71196	0	Uiso	1
N8	N	0.63268	0.61286	0.29169	0	Uiso	1
C9	C	0.65696	0.68496	0.59835	0	Uiso	1
C10	C	0.15302	0.42646	0.71316	0	Uiso	1
C11	C	0.21648	0.45086	0.71316	0	Uiso	1
C12	C	0.24609	0.50786	0.71318	0	Uiso	1
C13	C	0.26504	0.52002	0.71321	0	Uiso	1
N14	N	0.28514	0.56624	0.92535	0	Uiso	1
C15	C	0.29693	0.57359	0.96055	0	Uiso	1
C16	C	0.24661	0.58001	1.02699	0	Uiso	1
C17	C	0.27506	0.59372	0.10124	0	Uiso	1
C18	C	0.6549	0.61023	0.13586	0	Uiso	1
C19	C	0.61401	0.60516	0.06666	0	Uiso	1
C20	C	0.62426	0.58887	0.99775	0	Uiso	1
C21	C	0.14425	0.11762	0.32721	0	Uiso	1
C22	C	0.10519	0.05824	0.66935	0	Uiso	1
C23	C	0.1714	0.05855	0.64033	0	Uiso	1
C24	C	0.2485	0.05217	0.6471	0	Uiso	1
C25	C	0.25893	0.04558	0.68297	0	Uiso	1
C26	C	0.19229	0.04526	0.71224	0	Uiso	1
N27	N	0.12149	0.13529	0.29209	0	Uiso	1
C28	C	0.15838	0.06586	0.60393	0	Uiso	1
C29	C	-0.14245	0.32406	0.71317	0	Uiso	1
C30	C	0.81556	0.29882	0.71316	0	Uiso	1
C31	C	0.84814	0.24197	0.71322	0	Uiso	1
C32	C	0.84103	0.22958	0.7133	0	Uiso	1
N33	N	0.79111	0.17636	0.91177	0	Uiso	1
C34	C	0.79885	0.17028	0.95001	0	Uiso	1
C35	C	0.87169	0.16706	1.0148	0	Uiso	1
C36	C	0.88637	0.1545	0.09163	0	Uiso	1
C37	C	0.15433	0.13804	0.13561	0	Uiso	1
C38	C	0.23261	0.14163	0.07237	0	Uiso	1
C39	C	0.25348	0.15514	0.99673	0	Uiso	1

C40	C	0.61537	0.55163	0.79505	0	Uiso	1
O41	O	0.892	0.14711	0.28729	0	Uiso	1
O42	O	0.87946	0.10099	0.28738	0	Uiso	1
C43	C	0.11636	0.41508	0.78685	0	Uiso	1
C44	C	0.24162	0.46282	0.78686	0	Uiso	1
C45	C	0.80319	0.2865	0.78686	0	Uiso	1
C46	C	0.88737	0.33581	0.78684	0	Uiso	1
C47	C	0.86651	0.08167	0.53661	0	Uiso	1
C48	C	0.81588	0.08813	0.47007	0	Uiso	1
C49	C	0.8257	0.09577	0.43364	0	Uiso	1
C50	C	0.89556	0.11234	0.39888	0	Uiso	1
C51	C	0.73712	0.07283	0.50749	0	Uiso	1
C52	C	0.78484	0.10087	0.36429	0	Uiso	1
C53	C	0.81258	0.15799	0.02469	0	Uiso	1
C54	C	0.8216	0.15135	0.0635	0	Uiso	1
C55	C	0.89808	0.14793	0.12729	0	Uiso	1
C56	C	0.77921	0.13507	0.10813	0	Uiso	1
C57	C	0.86497	0.17315	-0.02211	0	Uiso	1
C58	C	0.73975	0.16125	-0.04029	0	Uiso	1
H59	H	0.77631	0.69176	0.72864	0	Uiso	1
H60	H	0.77235	0.68054	0.66143	0	Uiso	1
H61	H	0.55475	0.70299	0.62083	0	Uiso	1
H62	H	0.55829	0.714	0.68792	0	Uiso	1
H63	H	0.60478	0.68761	0.57539	0	Uiso	1
H64	H	0.16864	0.43106	0.6831	0	Uiso	1
H65	H	0.20661	0.44597	0.6831	0	Uiso	1
H66	H	0.23828	0.50291	0.68309	0	Uiso	1
H67	H	0.27333	0.52497	0.68315	0	Uiso	1
H68	H	0.19745	0.57924	1.05287	0	Uiso	1
H69	H	0.22547	0.58618	0.10156	0	Uiso	1
H70	H	0.57234	0.60699	0.03861	0	Uiso	1
H71	H	0.56756	0.5954	0.99955	0	Uiso	1
H72	H	0.04233	0.0634	0.66401	0	Uiso	1
H73	H	0.30272	0.05234	0.6234	0	Uiso	1
H74	H	0.32176	0.04043	0.68854	0	Uiso	1
H75	H	0.09691	0.07146	0.60065	0	Uiso	1
H76	H	-0.15552	0.31941	0.68293	0	Uiso	1
H77	H	0.82002	0.30377	0.68291	0	Uiso	1
H78	H	0.85121	0.24701	0.68312	0	Uiso	1
H79	H	0.83756	0.22451	0.68326	0	Uiso	1
H80	H	0.92583	0.16952	1.03701	0	Uiso	1

H81	H	0.93008	0.1625	0.08548	0	Uiso	1
H82	H	0.28679	0.13904	0.05036	0	Uiso	1
H83	H	0.30221	0.1478	1.00411	0	Uiso	1
H84	H	0.56039	0.55143	0.77173	0	Uiso	1
H85	H	0.10154	0.41044	0.81695	0	Uiso	1
H86	H	0.2524	0.46766	0.81695	0	Uiso	1
H87	H	0.79743	0.2815	0.81695	0	Uiso	1
H88	H	0.89915	0.3406	0.81695	0	Uiso	1
H89	H	0.91579	0.08236	0.56247	0	Uiso	1
H90	H	0.94473	0.11994	0.39914	0	Uiso	1
H91	H	0.68029	0.06633	0.50946	0	Uiso	1
H92	H	0.74344	0.09906	0.33615	0	Uiso	1
H93	H	0.95098	0.15069	0.14974	0	Uiso	1
H94	H	0.73426	0.12723	0.11486	0	Uiso	1
H95	H	0.91358	0.1805	-0.02957	0	Uiso	1
H96	H	0.6857	0.15882	-0.0626	0	Uiso	1
N97	N	0.58274	0.88783	0.75	0	Uiso	1
C98	C	0.64102	0.7611	0.75	0	Uiso	1
N99	N	0.16953	0.21017	0.75	0	Uiso	1
C100	C	0.70212	0.78031	0.75	0	Uiso	1
C101	C	0.59723	0.84169	0.75	0	Uiso	1
N102	N	0.71004	0.76769	0.75	0	Uiso	1
C103	C	0.66199	0.72311	0.75	0	Uiso	1
C104	C	0.64852	0.74817	0.75	0	Uiso	1
C105	C	0.54736	0.89826	0.75	0	Uiso	1
N106	N	0.41716	0.86329	0.75	0	Uiso	1
C107	C	0.28503	-0.01087	0.75	0	Uiso	1
N108	N	0.70394	0.53887	0.75	0	Uiso	1
C109	C	0.24646	-0.03116	0.75	0	Uiso	1
C110	C	0.40216	0.90937	0.75	0	Uiso	1
N111	N	0.2243	-0.01885	0.75	0	Uiso	1
C112	C	0.22584	0.02632	0.75	0	Uiso	1
C113	C	0.26328	0.00177	0.75	0	Uiso	1
C114	C	0.45264	0.85287	0.75	0	Uiso	1
C115	C	0.17098	0.43233	0.75	0	Uiso	1
C116	C	0.20315	0.44477	0.75	0	Uiso	1
C117	C	0.8225	0.30512	0.75	0	Uiso	1
C118	C	0.84366	0.31799	0.75	0	Uiso	1
H119	H	0.608	0.35289	0.75	0	Uiso	1
H120	H	0.62054	0.39901	0.75	0	Uiso	1
H121	H	0.8296	0.37555	0.75	0	Uiso	1

Ni122	Ni	0.5	0.87556	0.75	0	Uiso	1
Ni123	Ni	0.62504	0.62407	0.25	0	Uiso	1
O124	O	0.17034	0.61349	0.25	0	Uiso	1
O125	O	0.17045	0.63541	0.25	0	Uiso	1

Table S2. Fractional atomistic coordinates for Pawley-refined unit cell parameters of 3D-Ni-N_4-COF with pto topology. Space group P1, a = 52.5067 Å, b = 52.5072 Å, c = 52.5074 Å, $\alpha = \gamma = \beta$ = 90°.

C1	C	0.93543	0.53239	0.68254	0	Uiso	1
C2	C	0.9366	0.55539	0.669	0	Uiso	1
C3	C	0.91572	0.57185	0.66836	0	Uiso	1
C4	C	0.89312	0.56564	0.68118	0	Uiso	1
C5	C	0.89189	0.54256	0.69462	0	Uiso	1
C6	C	0.91285	0.52618	0.6953	0	Uiso	1
C7	C	0.8711	0.5833	0.68077	0	Uiso	1
C8	C	0.84759	0.57607	0.69128	0	Uiso	1
C9	C	0.82695	0.5929	0.69113	0	Uiso	1
C10	C	0.82958	0.61725	0.68052	0	Uiso	1
C11	C	0.85291	0.62455	0.67002	0	Uiso	1
C12	C	0.8735	0.60771	0.6701	0	Uiso	1
C13	C	0.80844	0.63549	0.68048	0	Uiso	1
N14	N	0.78597	0.62929	0.68862	0	Uiso	1
C15	C	0.72344	0.68061	0.69155	0	Uiso	1
C16	C	0.74766	0.68941	0.68443	0	Uiso	1
C17	C	0.7683	0.67268	0.68323	0	Uiso	1
C18	C	0.76508	0.64682	0.68926	0	Uiso	1
C19	C	0.74102	0.63801	0.69646	0	Uiso	1
C20	C	0.72034	0.65474	0.69757	0	Uiso	1
C21	C	0.07648	0.46496	0.68296	0	Uiso	1
C22	C	0.0755	0.4419	0.66951	0	Uiso	1
C23	C	0.09643	0.42549	0.66916	0	Uiso	1
C24	C	0.11888	0.43181	0.68221	0	Uiso	1
C25	C	0.11989	0.45492	0.69559	0	Uiso	1
C26	C	0.09888	0.47125	0.69598	0	Uiso	1
C27	C	0.14095	0.41422	0.68213	0	Uiso	1
C28	C	0.16439	0.42167	0.69268	0	Uiso	1
C29	C	0.18507	0.4049	0.69291	0	Uiso	1
C30	C	0.18257	0.3804	0.68265	0	Uiso	1
C31	C	0.15933	0.37287	0.6721	0	Uiso	1
C32	C	0.13869	0.38966	0.67179	0	Uiso	1
C33	C	0.20368	0.36214	0.68319	0	Uiso	1
N34	N	0.22613	0.36848	0.6913	0	Uiso	1
C35	C	0.28809	0.31665	0.69674	0	Uiso	1
C36	C	0.26385	0.3079	0.68961	0	Uiso	1
C37	C	0.24343	0.3248	0.68754	0	Uiso	1
C38	C	0.24688	0.35082	0.69263	0	Uiso	1
C39	C	0.271	0.35965	0.69966	0	Uiso	1

C40	C	0.29147	0.34273	0.70163	0	Uiso	1
C41	C	0.07247	0.52435	0.31815	0	Uiso	1
C42	C	0.07893	0.53664	0.34089	0	Uiso	1
C43	C	0.10064	0.552	0.34211	0	Uiso	1
C44	C	0.11618	0.55528	0.32059	0	Uiso	1
C45	C	0.1095	0.54298	0.29781	0	Uiso	1
C46	C	0.08768	0.52781	0.29654	0	Uiso	1
C47	C	0.13878	0.5721	0.3216	0	Uiso	1
C48	C	0.15777	0.57049	0.30285	0	Uiso	1
C49	C	0.17872	0.58689	0.30338	0	Uiso	1
C50	C	0.18089	0.60527	0.3226	0	Uiso	1
C51	C	0.16208	0.60695	0.34135	0	Uiso	1
C52	C	0.14122	0.59041	0.34094	0	Uiso	1
C53	C	0.20211	0.62342	0.32294	0	Uiso	1
N54	N	0.22176	0.62053	0.30827	0	Uiso	1
C55	C	0.28261	0.67374	0.30295	0	Uiso	1
C56	C	0.25991	0.68042	0.31546	0	Uiso	1
C57	C	0.23995	0.66297	0.31766	0	Uiso	1
C58	C	0.24227	0.63847	0.30723	0	Uiso	1
C59	C	0.26477	0.63169	0.29474	0	Uiso	1
C60	C	0.2848	0.6491	0.29274	0	Uiso	1
C61	C	0.9262	0.47228	0.3195	0	Uiso	1
C62	C	0.92029	0.4602	0.34252	0	Uiso	1
C63	C	0.89849	0.44505	0.34447	0	Uiso	1
C64	C	0.8823	0.44178	0.32343	0	Uiso	1
C65	C	0.88846	0.4538	0.30037	0	Uiso	1
C66	C	0.91037	0.46874	0.29835	0	Uiso	1
C67	C	0.85952	0.42531	0.32526	0	Uiso	1
C68	C	0.83949	0.42786	0.30774	0	Uiso	1
C69	C	0.81827	0.41185	0.30905	0	Uiso	1
C70	C	0.81689	0.39293	0.32781	0	Uiso	1
C71	C	0.83677	0.39029	0.34532	0	Uiso	1
C72	C	0.85789	0.40645	0.34415	0	Uiso	1
C73	C	0.79539	0.37516	0.32888	0	Uiso	1
N74	N	0.77471	0.37918	0.316	0	Uiso	1
C75	C	0.71272	0.32718	0.31267	0	Uiso	1
C76	C	0.73644	0.31928	0.32221	0	Uiso	1
C77	C	0.7568	0.33632	0.32377	0	Uiso	1
C78	C	0.75387	0.36161	0.31563	0	Uiso	1
C79	C	0.73035	0.36958	0.30605	0	Uiso	1
C80	C	0.70991	0.35258	0.30473	0	Uiso	1

C81	C	0.68418	0.92863	0.52558	0	Uiso	1
C82	C	0.66143	0.92217	0.53787	0	Uiso	1
C83	C	0.66022	0.90046	0.55323	0	Uiso	1
C84	C	0.68174	0.88492	0.55651	0	Uiso	1
C85	C	0.70452	0.89161	0.54421	0	Uiso	1
C86	C	0.70579	0.91342	0.52904	0	Uiso	1
C87	C	0.68073	0.86232	0.57333	0	Uiso	1
C88	C	0.69948	0.84333	0.57172	0	Uiso	1
C89	C	0.69895	0.82238	0.58812	0	Uiso	1
C90	C	0.67973	0.82021	0.6065	0	Uiso	1
C91	C	0.66098	0.83902	0.60818	0	Uiso	1
C92	C	0.66139	0.85988	0.59165	0	Uiso	1
C93	C	0.67939	0.799	0.62465	0	Uiso	1
N94	N	0.69406	0.77935	0.62176	0	Uiso	1
C95	C	0.69937	0.71849	0.67497	0	Uiso	1
C96	C	0.68687	0.74119	0.68165	0	Uiso	1
C97	C	0.68467	0.76115	0.6642	0	Uiso	1
C98	C	0.6951	0.75883	0.6397	0	Uiso	1
C99	C	0.70759	0.73634	0.63292	0	Uiso	1
C100	C	0.70959	0.7163	0.65032	0	Uiso	1
C101	C	0.68283	0.0749	0.4735	0	Uiso	1
C102	C	0.65981	0.08081	0.46143	0	Uiso	1
C103	C	0.65786	0.10261	0.44628	0	Uiso	1
C104	C	0.67889	0.11879	0.443	0	Uiso	1
C105	C	0.70196	0.11264	0.45503	0	Uiso	1
C106	C	0.70398	0.09073	0.46997	0	Uiso	1
C107	C	0.67706	0.14157	0.42654	0	Uiso	1
C108	C	0.69459	0.16161	0.42909	0	Uiso	1
C109	C	0.69327	0.18282	0.41308	0	Uiso	1
C110	C	0.67451	0.1842	0.39415	0	Uiso	1
C111	C	0.65701	0.16432	0.39151	0	Uiso	1
C112	C	0.65818	0.14321	0.40768	0	Uiso	1
C113	C	0.67345	0.20571	0.37639	0	Uiso	1
N114	N	0.68632	0.22639	0.38041	0	Uiso	1
C115	C	0.68965	0.28837	0.32841	0	Uiso	1
C116	C	0.68011	0.26465	0.32051	0	Uiso	1
C117	C	0.67856	0.24429	0.33754	0	Uiso	1
C118	C	0.6867	0.24723	0.36283	0	Uiso	1
C119	C	0.69627	0.27075	0.37081	0	Uiso	1
C120	C	0.69759	0.29119	0.35381	0	Uiso	1
C121	C	0.31978	0.06567	0.53361	0	Uiso	1

C122	C	0.33332	0.0645	0.55661	0	Uiso	1
C123	C	0.33397	0.08538	0.57307	0	Uiso	1
C124	C	0.32115	0.10799	0.56686	0	Uiso	1
C125	C	0.30771	0.10922	0.54378	0	Uiso	1
C126	C	0.30703	0.08825	0.5274	0	Uiso	1
C127	C	0.32156	0.13	0.58452	0	Uiso	1
C128	C	0.31105	0.15352	0.57729	0	Uiso	1
C129	C	0.3112	0.17415	0.59412	0	Uiso	1
C130	C	0.32182	0.17153	0.61847	0	Uiso	1
C131	C	0.33231	0.1482	0.62577	0	Uiso	1
C132	C	0.33223	0.12761	0.60893	0	Uiso	1
C133	C	0.32185	0.19266	0.63671	0	Uiso	1
N134	N	0.31372	0.21513	0.63051	0	Uiso	1
C135	C	0.31078	0.27767	0.68183	0	Uiso	1
C136	C	0.31791	0.25344	0.69063	0	Uiso	1
C137	C	0.3191	0.2328	0.6739	0	Uiso	1
C138	C	0.31307	0.23602	0.64804	0	Uiso	1
C139	C	0.30587	0.26009	0.63923	0	Uiso	1
C140	C	0.30477	0.28076	0.65595	0	Uiso	1
C141	C	0.31936	0.92463	0.46618	0	Uiso	1
C142	C	0.33281	0.92561	0.44312	0	Uiso	1
C143	C	0.33316	0.90468	0.42671	0	Uiso	1
C144	C	0.32012	0.88223	0.43303	0	Uiso	1
C145	C	0.30673	0.88122	0.45614	0	Uiso	1
C146	C	0.30634	0.90222	0.47247	0	Uiso	1
C147	C	0.32019	0.86015	0.41545	0	Uiso	1
C148	C	0.30964	0.83672	0.42289	0	Uiso	1
C149	C	0.30941	0.81604	0.40613	0	Uiso	1
C150	C	0.31967	0.81854	0.38162	0	Uiso	1
C151	C	0.33022	0.84178	0.3741	0	Uiso	1
C152	C	0.33053	0.86241	0.39089	0	Uiso	1
C153	C	0.31914	0.79742	0.36337	0	Uiso	1
N154	N	0.31102	0.77498	0.3697	0	Uiso	1
C155	C	0.30558	0.71302	0.31788	0	Uiso	1
C156	C	0.31271	0.73726	0.30912	0	Uiso	1
C157	C	0.31477	0.75768	0.32602	0	Uiso	1
C158	C	0.30969	0.75423	0.35205	0	Uiso	1
C159	C	0.30266	0.73011	0.36087	0	Uiso	1
C160	C	0.30069	0.70963	0.34396	0	Uiso	1
C161	C	0.53615	0.68175	0.92585	0	Uiso	1
C162	C	0.5592	0.66829	0.92684	0	Uiso	1

C163	C	0.57562	0.66795	0.90591	0	Uiso	1
C164	C	0.5693	0.68099	0.88346	0	Uiso	1
C165	C	0.54619	0.69438	0.88244	0	Uiso	1
C166	C	0.52986	0.69477	0.90345	0	Uiso	1
C167	C	0.58688	0.68092	0.86138	0	Uiso	1
C168	C	0.57944	0.69146	0.83795	0	Uiso	1
C169	C	0.5962	0.6917	0.81727	0	Uiso	1
C170	C	0.62071	0.68143	0.81977	0	Uiso	1
C171	C	0.62823	0.67089	0.84301	0	Uiso	1
C172	C	0.61144	0.67057	0.86364	0	Uiso	1
C173	C	0.63896	0.68197	0.79865	0	Uiso	1
N174	N	0.63263	0.69009	0.77621	0	Uiso	1
C175	C	0.68445	0.69553	0.71425	0	Uiso	1
C176	C	0.69321	0.68839	0.73849	0	Uiso	1
C177	C	0.67631	0.68633	0.7589	0	Uiso	1
C178	C	0.65028	0.69142	0.75546	0	Uiso	1
C179	C	0.64146	0.69845	0.73134	0	Uiso	1
C180	C	0.65837	0.70041	0.71086	0	Uiso	1
C181	C	0.46871	0.68133	0.0669	0	Uiso	1
C182	C	0.44571	0.66779	0.06573	0	Uiso	1
C183	C	0.42926	0.66714	0.08661	0	Uiso	1
C184	C	0.43546	0.67996	0.10921	0	Uiso	1
C185	C	0.45854	0.6934	0.11044	0	Uiso	1
C186	C	0.47493	0.69409	0.08948	0	Uiso	1
C187	C	0.4178	0.67956	0.13123	0	Uiso	1
C188	C	0.42503	0.69006	0.15474	0	Uiso	1
C189	C	0.4082	0.68991	0.17538	0	Uiso	1
C190	C	0.38385	0.6793	0.17275	0	Uiso	1
C191	C	0.37655	0.6688	0.14942	0	Uiso	1
C192	C	0.39339	0.66888	0.12883	0	Uiso	1
C193	C	0.36561	0.67926	0.19388	0	Uiso	1
N194	N	0.37181	0.68739	0.21636	0	Uiso	1
C195	C	0.32049	0.69032	0.27889	0	Uiso	1
C196	C	0.31169	0.6832	0.25467	0	Uiso	1
C197	C	0.32842	0.682	0.23403	0	Uiso	1
C198	C	0.35428	0.68804	0.23724	0	Uiso	1
C199	C	0.36309	0.69523	0.26131	0	Uiso	1
C200	C	0.34637	0.69634	0.28199	0	Uiso	1
C201	C	0.52882	0.31827	0.07612	0	Uiso	1
C202	C	0.5409	0.34129	0.08203	0	Uiso	1
C203	C	0.55605	0.34324	0.10383	0	Uiso	1

C204	C	0.55932	0.32221	0.12002	0	Uiso	1
C205	C	0.5473	0.29914	0.11386	0	Uiso	1
C206	C	0.53235	0.29713	0.09195	0	Uiso	1
C207	C	0.57579	0.32404	0.1428	0	Uiso	1
C208	C	0.57324	0.30651	0.16283	0	Uiso	1
C209	C	0.58925	0.30782	0.18405	0	Uiso	1
C210	C	0.60817	0.32659	0.18543	0	Uiso	1
C211	C	0.61081	0.34409	0.16555	0	Uiso	1
C212	C	0.59464	0.34292	0.14444	0	Uiso	1
C213	C	0.62593	0.32765	0.20694	0	Uiso	1
N214	N	0.62191	0.31477	0.22761	0	Uiso	1
C215	C	0.67392	0.31145	0.2896	0	Uiso	1
C216	C	0.68181	0.32098	0.26588	0	Uiso	1
C217	C	0.66478	0.32254	0.24552	0	Uiso	1
C218	C	0.63949	0.3144	0.24846	0	Uiso	1
C219	C	0.63151	0.30483	0.27198	0	Uiso	1
C220	C	0.64851	0.3035	0.29241	0	Uiso	1
C221	C	0.47675	0.31692	0.92985	0	Uiso	1
C222	C	0.46446	0.33967	0.92339	0	Uiso	1
C223	C	0.44911	0.34088	0.90168	0	Uiso	1
C224	C	0.44582	0.31937	0.88614	0	Uiso	1
C225	C	0.45812	0.29659	0.89283	0	Uiso	1
C226	C	0.47329	0.29531	0.91464	0	Uiso	1
C227	C	0.429	0.32038	0.86354	0	Uiso	1
C228	C	0.43062	0.30162	0.84455	0	Uiso	1
C229	C	0.41422	0.30215	0.8236	0	Uiso	1
C230	C	0.39584	0.32138	0.82143	0	Uiso	1
C231	C	0.39415	0.34013	0.84024	0	Uiso	1
C232	C	0.41069	0.33971	0.8611	0	Uiso	1
C233	C	0.37769	0.32171	0.80021	0	Uiso	1
N234	N	0.38057	0.30704	0.78056	0	Uiso	1
C235	C	0.32737	0.30173	0.71971	0	Uiso	1
C236	C	0.32068	0.31423	0.74241	0	Uiso	1
C237	C	0.33813	0.31643	0.76237	0	Uiso	1
C238	C	0.36263	0.306	0.76005	0	Uiso	1
C239	C	0.36941	0.29351	0.73756	0	Uiso	1
C240	C	0.35201	0.29151	0.71752	0	Uiso	1
C241	C	0.06983	0.46455	0.31684	0	Uiso	1
C242	C	0.06867	0.44155	0.33038	0	Uiso	1
C243	C	0.08954	0.42509	0.33102	0	Uiso	1
C244	C	0.11215	0.4313	0.3182	0	Uiso	1

C245	C	0.11338	0.45437	0.30477	0	Uiso	1
C246	C	0.09242	0.47076	0.30408	0	Uiso	1
C247	C	0.13416	0.41364	0.31861	0	Uiso	1
C248	C	0.15768	0.42086	0.3081	0	Uiso	1
C249	C	0.17831	0.40404	0.30825	0	Uiso	1
C250	C	0.17569	0.37969	0.31887	0	Uiso	1
C251	C	0.15236	0.37238	0.32936	0	Uiso	1
C252	C	0.13177	0.38923	0.32929	0	Uiso	1
C253	C	0.19682	0.36145	0.3189	0	Uiso	1
N254	N	0.2193	0.36765	0.31077	0	Uiso	1
C255	C	0.28183	0.31632	0.30784	0	Uiso	1
C256	C	0.25761	0.30752	0.31496	0	Uiso	1
C257	C	0.23697	0.32425	0.31616	0	Uiso	1
C258	C	0.24018	0.35011	0.31012	0	Uiso	1
C259	C	0.26425	0.35893	0.30293	0	Uiso	1
C260	C	0.28492	0.3422	0.30182	0	Uiso	1
C261	C	0.92879	0.53198	0.31642	0	Uiso	1
C262	C	0.92977	0.55504	0.32987	0	Uiso	1
C263	C	0.90884	0.57145	0.33022	0	Uiso	1
C264	C	0.88639	0.56513	0.31717	0	Uiso	1
C265	C	0.88538	0.54202	0.30379	0	Uiso	1
C266	C	0.90638	0.52569	0.3034	0	Uiso	1
C267	C	0.86432	0.58271	0.31725	0	Uiso	1
C268	C	0.84088	0.57527	0.3067	0	Uiso	1
C269	C	0.8202	0.59204	0.30647	0	Uiso	1
C270	C	0.8227	0.61654	0.31673	0	Uiso	1
C271	C	0.84594	0.62407	0.32728	0	Uiso	1
C272	C	0.86658	0.60728	0.32759	0	Uiso	1
C273	C	0.80159	0.6348	0.31619	0	Uiso	1
N274	N	0.77914	0.62846	0.30808	0	Uiso	1
C275	C	0.71719	0.68029	0.30264	0	Uiso	1
C276	C	0.74142	0.68904	0.30977	0	Uiso	1
C277	C	0.76184	0.67214	0.31184	0	Uiso	1
C278	C	0.75839	0.64612	0.30675	0	Uiso	1
C279	C	0.73427	0.63729	0.29972	0	Uiso	1
C280	C	0.7138	0.65421	0.29775	0	Uiso	1
C281	C	0.93279	0.47259	0.68123	0	Uiso	1
C282	C	0.92634	0.46029	0.65849	0	Uiso	1
C283	C	0.90462	0.44494	0.65727	0	Uiso	1
C284	C	0.88908	0.44166	0.67879	0	Uiso	1
C285	C	0.89577	0.45396	0.70157	0	Uiso	1

C286	C	0.91758	0.46913	0.70284	0	Uiso	1
C287	C	0.86649	0.42484	0.67778	0	Uiso	1
C288	C	0.8475	0.42645	0.69654	0	Uiso	1
C289	C	0.82654	0.41005	0.69601	0	Uiso	1
C290	C	0.82437	0.39167	0.67678	0	Uiso	1
C291	C	0.84319	0.38998	0.65803	0	Uiso	1
C292	C	0.86404	0.40652	0.65845	0	Uiso	1
C293	C	0.80316	0.37352	0.67645	0	Uiso	1
N294	N	0.78351	0.3764	0.69112	0	Uiso	1
C295	C	0.72266	0.3232	0.69643	0	Uiso	1
C296	C	0.74536	0.31651	0.68393	0	Uiso	1
C297	C	0.76531	0.33396	0.68173	0	Uiso	1
C298	C	0.763	0.35847	0.69216	0	Uiso	1
C299	C	0.7405	0.36525	0.70465	0	Uiso	1
C300	C	0.72047	0.34784	0.70665	0	Uiso	1
C301	C	0.07907	0.52466	0.67988	0	Uiso	1
C302	C	0.08498	0.53673	0.65686	0	Uiso	1
C303	C	0.10678	0.55189	0.65491	0	Uiso	1
C304	C	0.12296	0.55516	0.67595	0	Uiso	1
C305	C	0.11681	0.54314	0.69901	0	Uiso	1
C306	C	0.0949	0.52819	0.70103	0	Uiso	1
C307	C	0.14575	0.57163	0.67412	0	Uiso	1
C308	C	0.16578	0.56908	0.69164	0	Uiso	1
C309	C	0.18699	0.58509	0.69033	0	Uiso	1
C310	C	0.18837	0.60401	0.67157	0	Uiso	1
C311	C	0.1685	0.60665	0.65407	0	Uiso	1
C312	C	0.14738	0.59048	0.65523	0	Uiso	1
C313	C	0.20988	0.62177	0.67051	0	Uiso	1
N314	N	0.23056	0.61775	0.68338	0	Uiso	1
C315	C	0.29254	0.66976	0.68671	0	Uiso	1
C316	C	0.26882	0.67765	0.67718	0	Uiso	1
C317	C	0.24846	0.66062	0.67562	0	Uiso	1
C318	C	0.2514	0.63533	0.68376	0	Uiso	1
C319	C	0.27492	0.62735	0.69333	0	Uiso	1
C320	C	0.29536	0.64435	0.69465	0	Uiso	1
C321	C	0.32109	0.06831	0.47381	0	Uiso	1
C322	C	0.34384	0.07477	0.46152	0	Uiso	1
C323	C	0.34505	0.09648	0.44616	0	Uiso	1
C324	C	0.32353	0.11202	0.44288	0	Uiso	1
C325	C	0.30075	0.10533	0.45518	0	Uiso	1
C326	C	0.29948	0.08352	0.47035	0	Uiso	1

C327	C	0.32455	0.13462	0.42606	0	Uiso	1
C328	C	0.30579	0.15361	0.42767	0	Uiso	1
C329	C	0.30632	0.17456	0.41127	0	Uiso	1
C330	C	0.32554	0.17673	0.39289	0	Uiso	1
C331	C	0.34429	0.15791	0.3912	0	Uiso	1
C332	C	0.34388	0.13706	0.40774	0	Uiso	1
C333	C	0.32588	0.19794	0.37474	0	Uiso	1
N334	N	0.31121	0.21759	0.37762	0	Uiso	1
C335	C	0.30589	0.27844	0.32442	0	Uiso	1
C336	C	0.31839	0.25574	0.31773	0	Uiso	1
C337	C	0.3206	0.23579	0.33518	0	Uiso	1
C338	C	0.31017	0.2381	0.35969	0	Uiso	1
C339	C	0.29768	0.2606	0.36647	0	Uiso	1
C340	C	0.29568	0.28063	0.34906	0	Uiso	1
C341	C	0.32244	0.92204	0.52588	0	Uiso	1
C342	C	0.34546	0.91613	0.53796	0	Uiso	1
C343	C	0.34741	0.89433	0.55311	0	Uiso	1
C344	C	0.32638	0.87814	0.55638	0	Uiso	1
C345	C	0.30331	0.8843	0.54436	0	Uiso	1
C346	C	0.3013	0.90621	0.52942	0	Uiso	1
C347	C	0.32821	0.85536	0.57285	0	Uiso	1
C348	C	0.31068	0.83533	0.5703	0	Uiso	1
C349	C	0.312	0.81411	0.58631	0	Uiso	1
C350	C	0.33076	0.81274	0.60524	0	Uiso	1
C351	C	0.34826	0.83261	0.60787	0	Uiso	1
C352	C	0.34709	0.85373	0.59171	0	Uiso	1
C353	C	0.33182	0.79123	0.623	0	Uiso	1
N354	N	0.31895	0.77055	0.61898	0	Uiso	1
C355	C	0.31562	0.70856	0.67098	0	Uiso	1
C356	C	0.32515	0.73228	0.67888	0	Uiso	1
C357	C	0.32671	0.75264	0.66184	0	Uiso	1
C358	C	0.31857	0.74971	0.63655	0	Uiso	1
C359	C	0.309	0.72619	0.62858	0	Uiso	1
C360	C	0.30768	0.70575	0.64558	0	Uiso	1
C361	C	0.68549	0.93127	0.46578	0	Uiso	1
C362	C	0.67195	0.93243	0.44278	0	Uiso	1
C363	C	0.6713	0.91156	0.42632	0	Uiso	1
C364	C	0.68413	0.88895	0.43253	0	Uiso	1
C365	C	0.69756	0.88772	0.4556	0	Uiso	1
C366	C	0.69825	0.90868	0.47199	0	Uiso	1
C367	C	0.68372	0.86694	0.41487	0	Uiso	1

C368	C	0.69423	0.84342	0.42209	0	Uiso	1
C369	C	0.69408	0.82279	0.40527	0	Uiso	1
C370	C	0.68346	0.82541	0.38092	0	Uiso	1
C371	C	0.67296	0.84874	0.37361	0	Uiso	1
C372	C	0.67304	0.86933	0.39046	0	Uiso	1
C373	C	0.68342	0.80428	0.36268	0	Uiso	1
N374	N	0.69156	0.78181	0.36888	0	Uiso	1
C375	C	0.69449	0.71927	0.31755	0	Uiso	1
C376	C	0.68737	0.7435	0.30875	0	Uiso	1
C377	C	0.68617	0.76414	0.32548	0	Uiso	1
C378	C	0.6922	0.76092	0.35134	0	Uiso	1
C379	C	0.6994	0.73685	0.36016	0	Uiso	1
C380	C	0.7005	0.71618	0.34343	0	Uiso	1
C381	C	0.68591	0.07231	0.53321	0	Uiso	1
C382	C	0.67246	0.07133	0.55626	0	Uiso	1
C383	C	0.67211	0.09226	0.57268	0	Uiso	1
C384	C	0.68516	0.11471	0.56636	0	Uiso	1
C385	C	0.69854	0.11572	0.54325	0	Uiso	1
C386	C	0.69893	0.09472	0.52692	0	Uiso	1
C387	C	0.68508	0.13678	0.58394	0	Uiso	1
C388	C	0.69563	0.16022	0.57649	0	Uiso	1
C389	C	0.69586	0.1809	0.59326	0	Uiso	1
C390	C	0.6856	0.1784	0.61777	0	Uiso	1
C391	C	0.67505	0.15516	0.62529	0	Uiso	1
C392	C	0.67474	0.13452	0.6085	0	Uiso	1
C393	C	0.68614	0.19951	0.63602	0	Uiso	1
N394	N	0.69425	0.22196	0.62968	0	Uiso	1
C395	C	0.69969	0.28392	0.68151	0	Uiso	1
C396	C	0.69256	0.25968	0.69027	0	Uiso	1
C397	C	0.69049	0.23926	0.67337	0	Uiso	1
C398	C	0.69558	0.24271	0.64734	0	Uiso	1
C399	C	0.70261	0.26683	0.63852	0	Uiso	1
C400	C	0.70458	0.2873	0.65543	0	Uiso	1
C401	C	0.46912	0.31519	0.07353	0	Uiso	1
C402	C	0.44606	0.32864	0.07255	0	Uiso	1
C403	C	0.42965	0.32899	0.09348	0	Uiso	1
C404	C	0.43597	0.31595	0.11593	0	Uiso	1
C405	C	0.45908	0.30256	0.11694	0	Uiso	1
C406	C	0.47541	0.30217	0.09594	0	Uiso	1
C407	C	0.41838	0.31602	0.138	0	Uiso	1
C408	C	0.42583	0.30547	0.16144	0	Uiso	1

C409	C	0.40906	0.30524	0.18212	0	Uiso	1
C410	C	0.38456	0.3155	0.17962	0	Uiso	1
C411	C	0.37703	0.32605	0.15638	0	Uiso	1
C412	C	0.39382	0.32636	0.13574	0	Uiso	1
C413	C	0.3663	0.31497	0.20073	0	Uiso	1
N414	N	0.37264	0.30685	0.22318	0	Uiso	1
C415	C	0.32081	0.30141	0.28514	0	Uiso	1
C416	C	0.31206	0.30854	0.2609	0	Uiso	1
C417	C	0.32896	0.31061	0.24048	0	Uiso	1
C418	C	0.35498	0.30552	0.24393	0	Uiso	1
C419	C	0.36381	0.29849	0.26805	0	Uiso	1
C420	C	0.34689	0.29652	0.28852	0	Uiso	1
C421	C	0.53655	0.31561	0.93249	0	Uiso	1
C422	C	0.55955	0.32915	0.93366	0	Uiso	1
C423	C	0.57601	0.3298	0.91278	0	Uiso	1
C424	C	0.5698	0.31698	0.89018	0	Uiso	1
C425	C	0.54673	0.30354	0.88894	0	Uiso	1
C426	C	0.53034	0.30286	0.90991	0	Uiso	1
C427	C	0.58746	0.31738	0.86816	0	Uiso	1
C428	C	0.58024	0.30687	0.84464	0	Uiso	1
C429	C	0.59707	0.30703	0.82401	0	Uiso	1
C430	C	0.62141	0.31764	0.82664	0	Uiso	1
C431	C	0.62872	0.32814	0.84997	0	Uiso	1
C432	C	0.61187	0.32806	0.87056	0	Uiso	1
C433	C	0.63965	0.31768	0.8055	0	Uiso	1
N434	N	0.63346	0.30955	0.78303	0	Uiso	1
C435	C	0.68478	0.30661	0.7205	0	Uiso	1
C436	C	0.69358	0.31373	0.74472	0	Uiso	1
C437	C	0.67685	0.31493	0.76536	0	Uiso	1
C438	C	0.65099	0.3089	0.76214	0	Uiso	1
C439	C	0.64217	0.3017	0.73808	0	Uiso	1
C440	C	0.6589	0.3006	0.7174	0	Uiso	1
C441	C	0.47644	0.67866	0.92326	0	Uiso	1
C442	C	0.46437	0.65564	0.91735	0	Uiso	1
C443	C	0.44922	0.65369	0.89555	0	Uiso	1
C444	C	0.44594	0.67473	0.87937	0	Uiso	1
C445	C	0.45797	0.6978	0.88553	0	Uiso	1
C446	C	0.47291	0.69981	0.90744	0	Uiso	1
C447	C	0.42948	0.6729	0.85659	0	Uiso	1
C448	C	0.43203	0.69042	0.83655	0	Uiso	1
C449	C	0.41602	0.68911	0.81534	0	Uiso	1

C450	C	0.39709	0.67035	0.81396	0	Uiso	1
C451	C	0.39446	0.65285	0.83383	0	Uiso	1
C452	C	0.41062	0.65401	0.85495	0	Uiso	1
C453	C	0.37933	0.66929	0.79245	0	Uiso	1
N454	N	0.38335	0.68216	0.77177	0	Uiso	1
C455	C	0.33135	0.68549	0.70979	0	Uiso	1
C456	C	0.32345	0.67595	0.73351	0	Uiso	1
C457	C	0.34049	0.67439	0.75387	0	Uiso	1
C458	C	0.36578	0.68254	0.75093	0	Uiso	1
C459	C	0.37376	0.69211	0.72741	0	Uiso	1
C460	C	0.35675	0.69343	0.70697	0	Uiso	1
C461	C	0.52851	0.68002	0.06954	0	Uiso	1
C462	C	0.54081	0.65727	0.07599	0	Uiso	1
C463	C	0.55616	0.65606	0.09771	0	Uiso	1
C464	C	0.55944	0.67758	0.11325	0	Uiso	1
C465	C	0.54715	0.70036	0.10656	0	Uiso	1
C466	C	0.53197	0.70163	0.08475	0	Uiso	1
C467	C	0.57626	0.67656	0.13585	0	Uiso	1
C468	C	0.57465	0.69532	0.15484	0	Uiso	1
C469	C	0.59105	0.69479	0.17579	0	Uiso	1
C470	C	0.60943	0.67556	0.17796	0	Uiso	1
C471	C	0.61112	0.65681	0.15914	0	Uiso	1
C472	C	0.59458	0.65723	0.13829	0	Uiso	1
C473	C	0.62758	0.67523	0.19917	0	Uiso	1
N474	N	0.6247	0.6899	0.21882	0	Uiso	1
C475	C	0.6779	0.69521	0.27967	0	Uiso	1
C476	C	0.68459	0.68271	0.25697	0	Uiso	1
C477	C	0.66714	0.68051	0.23702	0	Uiso	1
C478	C	0.64263	0.69094	0.23933	0	Uiso	1
C479	C	0.63585	0.70343	0.26183	0	Uiso	1
C480	C	0.65326	0.70543	0.28186	0	Uiso	1
N481	N	0.3135	0.68761	0.68883	0	Uiso	1
N482	N	0.70215	0.30491	0.69918	0	Uiso	1
N483	N	0.69619	0.69796	0.30018	0	Uiso	1
N484	N	0.30314	0.29895	0.30613	0	Uiso	1
N485	N	0.69177	0.30933	0.31055	0	Uiso	1
N486	N	0.30312	0.69202	0.30021	0	Uiso	1
N487	N	0.30908	0.29898	0.6992	0	Uiso	1
N488	N	0.70212	0.69798	0.69325	0	Uiso	1
O489	O	0.02628	0.44859	0.69069	0.0608	Uani	1
O490	O	0.03131	0.5467	0.67838	0.05932	Uani	1

N491	N	0.03082	0.47321	0.68445	0.04881	Uani	1
N492	N	0.03328	0.52132	0.6826	0.04984	Uani	1
C493	C	0.05389	0.48207	0.68342	0.04846	Uani	1
C494	C	0.05522	0.51004	0.68238	0.04912	Uani	1
Ni495	Ni	0.00595	0.49865	0.68375	0.04629	Uani	1
O496	O	0.55252	0.68947	0.97605	0.0608	Uani	1
O497	O	0.4544	0.67716	0.97102	0.05932	Uani	1
N498	N	0.5279	0.68324	0.97151	0.04881	Uani	1
N499	N	0.47978	0.68138	0.96906	0.04984	Uani	1
C500	C	0.51904	0.68221	0.94844	0.04846	Uani	1
C501	C	0.49107	0.68116	0.94711	0.04912	Uani	1
Ni502	Ni	0.50246	0.68254	0.99638	0.04629	Uani	1
O503	O	0.69288	0.98155	0.44936	0.0608	Uani	1
O504	O	0.68304	0.97632	0.54771	0.05932	Uani	1
N505	N	0.68701	0.97693	0.47406	0.04881	Uani	1
N506	N	0.68638	0.97443	0.5222	0.04984	Uani	1
C507	C	0.68626	0.95384	0.48291	0.04846	Uani	1
C508	C	0.68595	0.9525	0.5109	0.04912	Uani	1
Ni509	Ni	0.6867	1.00178	0.49952	0.04629	Uani	1
O510	O	0.31164	0.97482	0.44981	0.0608	Uani	1
O511	O	0.32394	0.96979	0.54792	0.05932	Uani	1
N512	N	0.31787	0.97029	0.47443	0.04881	Uani	1
N513	N	0.31973	0.96783	0.52255	0.04984	Uani	1
C514	C	0.3189	0.94721	0.48329	0.04846	Uani	1
C515	C	0.31995	0.94589	0.51126	0.04912	Uani	1
Ni516	Ni	0.31857	0.99516	0.49987	0.04629	Uani	1
O517	O	0.98571	0.5488	0.68993	0.0608	Uani	1
O518	O	0.98048	0.45046	0.68009	0.05932	Uani	1
N519	N	0.9811	0.52411	0.68407	0.04881	Uani	1
N520	N	0.97859	0.47597	0.68343	0.04984	Uani	1
C521	C	0.95801	0.51526	0.68332	0.04846	Uani	1
C522	C	0.95666	0.48727	0.683	0.04912	Uani	1
O523	O	0.97899	0.54835	0.30869	0.0608	Uani	1
O524	O	0.97395	0.45024	0.321	0.05932	Uani	1
N525	N	0.97445	0.52373	0.31492	0.04881	Uani	1
N526	N	0.97199	0.47561	0.31678	0.04984	Uani	1
C527	C	0.95137	0.51487	0.31595	0.04846	Uani	1
C528	C	0.95005	0.4869	0.317	0.04912	Uani	1
O529	O	0.01955	0.44814	0.30945	0.0608	Uani	1
O530	O	0.02478	0.54648	0.31929	0.05932	Uani	1
N531	N	0.02417	0.47283	0.31531	0.04881	Uani	1

N532	N	0.02667	0.52097	0.31595	0.04984	Uani	1
C533	C	0.04726	0.48168	0.31606	0.04846	Uani	1
C534	C	0.0486	0.50967	0.31638	0.04912	Uani	1
Ni535	Ni	-0.00068	0.49829	0.31563	0.04629	Uani	1
O536	O	0.69364	0.02211	0.54958	0.0608	Uani	1
O537	O	0.68133	0.02715	0.45146	0.05932	Uani	1
N538	N	0.6874	0.02665	0.52496	0.04881	Uani	1
N539	N	0.68555	0.02911	0.47684	0.04984	Uani	1
C540	C	0.68637	0.04973	0.5161	0.04846	Uani	1
C541	C	0.68533	0.05105	0.48813	0.04912	Uani	1
O542	O	0.31239	0.01539	0.55002	0.0608	Uani	1
O543	O	0.32224	0.02062	0.45168	0.05932	Uani	1
N544	N	0.31826	0.02	0.52533	0.04881	Uani	1
N545	N	0.31889	0.02251	0.47719	0.04984	Uani	1
C546	C	0.31901	0.0431	0.51648	0.04846	Uani	1
C547	C	0.31932	0.04444	0.48849	0.04912	Uani	1
O548	O	0.55296	0.30822	0.98277	0.0608	Uani	1
O549	O	0.45462	0.31807	0.97754	0.05932	Uani	1
N550	N	0.52827	0.31409	0.97816	0.04881	Uani	1
N551	N	0.48013	0.31472	0.97565	0.04984	Uani	1
C552	C	0.51942	0.31484	0.95506	0.04846	Uani	1
C553	C	0.49143	0.31515	0.95372	0.04912	Uani	1
Ni554	Ni	0.50281	0.3144	1.003	0.04629	Uani	1
O555	O	0.45275	0.30747	0.02333	0.0608	Uani	1
O556	O	0.55086	0.31977	0.02837	0.05932	Uani	1
N557	N	0.47737	0.3137	0.02787	0.04881	Uani	1
N558	N	0.52549	0.31556	0.03033	0.04984	Uani	1
C559	C	0.48623	0.31473	0.05095	0.04846	Uani	1
C560	C	0.5142	0.31578	0.05227	0.04912	Uani	1
O561	O	0.4523	0.68872	0.01662	0.0608	Uani	1
O562	O	0.55065	0.67887	0.02185	0.05932	Uani	1
N563	N	0.477	0.68285	0.02123	0.04881	Uani	1
N564	N	0.52513	0.68222	0.02374	0.04984	Uani	1
C565	C	0.48584	0.6821	0.04432	0.04846	Uani	1
C566	C	0.51383	0.68179	0.04567	0.04912	Uani	1

Table S3. Fractional atomistic coordinates for Pawley-refined unit cell parameters of 2D-Ni-N_4-COF with AA stacking. Space group P222, a = 23.7722 Å, b = 3.8343 Å, c = 33.7078 Å, $\alpha = \gamma = \beta$ = 90°.

C1	C	0.66934	0.90827	0.78282	0	Uiso	1
C2	C	0.67788	0.79889	0.712	0	Uiso	1
C3	C	0.75626	1.04614	0.74806	0	Uiso	1
O4	O	0.60676	0.87627	0.96562	0	Uiso	1
C5	C	0.1535	0.95626	0.46331	0	Uiso	1
C6	C	0.20579	1.21574	0.5364	0	Uiso	1
C7	C	0.23759	1.25054	0.57101	0	Uiso	1
C8	C	0.21751	1.11672	0.60681	0	Uiso	1
C9	C	0.1655	0.9457	0.60771	0	Uiso	1
C10	C	0.13415	0.90767	0.57294	0	Uiso	1
C11	C	0.25189	0.84605	0.35736	0	Uiso	1
C12	C	0.46881	0.98761	0.0757	0	Uiso	1
N13	N	0.55317	1.01504	0.04037	0	Uiso	1
C14	C	0.56719	1.04772	0.11177	0	Uiso	1
C15	C	0.65502	0.92323	0.14613	0	Uiso	1
C16	C	0.72469	0.96391	0.217	0	Uiso	1
C17	C	0.73308	1.07087	0.28763	0	Uiso	1
C18	C	0.64635	1.21139	0.25305	0	Uiso	1
C19	C	0.58148	1.2408	0.17978	0	Uiso	1
N20	N	0.23413	1.04627	0.67687	0	Uiso	1
C21	C	0.6351	0.91165	0.81951	0	Uiso	1
C22	C	0.62136	1.09775	0.88773	0	Uiso	1
C23	C	0.54832	0.77638	0.85436	0	Uiso	1
C24	C	0.97065	1.00149	0.4645	0	Uiso	1
H25	H	0.65862	0.70101	0.6831	0	Uiso	1
H26	H	0.80118	1.15019	0.74858	0	Uiso	1
H27	H	0.22208	1.32768	0.50726	0	Uiso	1
H28	H	0.28004	1.3885	0.57005	0	Uiso	1
H29	H	0.14866	0.83712	0.63687	0	Uiso	1
H30	H	0.09214	0.76441	0.57366	0	Uiso	1
H31	H	0.29525	0.72016	0.35923	0	Uiso	1
H32	H	0.69901	0.80485	0.1457	0	Uiso	1
H33	H	0.74427	0.86706	0.18816	0	Uiso	1
H34	H	0.60161	1.31807	0.25382	0	Uiso	1
H35	H	0.56466	1.38005	0.20721	0	Uiso	1
H36	H	0.63779	1.23436	0.91545	0	Uiso	1
H37	H	0.50534	0.64456	0.85439	0	Uiso	1
H38	H	0.94778	1.0052	0.43478	0	Uiso	1
Ni39	Ni	0.5	1	1	0	Uiso	1

N40	N	0.12181	1	0.5	0	Uiso	1
C41	C	0.93979	1	0.5	0	Uiso	1
H42	H	1.60676	1	1	0	Uiso	1

Table S4. Fractional atomistic coordinates for Pawley-refined unit cell parameters of 2D-Ni-N_4-COF with AB stacking. Space group P1, a = 32.9727 Å, b = 24.8991 Å, c = 7.5942 Å, $\alpha = \gamma = \beta$ = 90°.

Ni1	Ni	0.49847	1.00232	0.24981	0	Uiso	1
N2	N	0.45542	0.95443	0.22936	0	Uiso	1
C3	C	0.41998	0.97751	0.229	0	Uiso	1
C4	C	0.42191	1.03647	0.25963	0	Uiso	1
N5	N	0.45853	1.05422	0.28442	0	Uiso	1
N6	N	0.53842	0.95043	0.21527	0	Uiso	1
C7	C	0.57503	0.96817	0.24013	0	Uiso	1
C8	C	0.57696	1.02713	0.27077	0	Uiso	1
N9	N	0.54153	1.05021	0.27033	0	Uiso	1
C10	C	0.38888	1.12602	0.20996	0	Uiso	1
C11	C	0.35559	1.16074	0.21918	0	Uiso	1
C12	C	0.31885	1.14329	0.29274	0	Uiso	1
C13	C	0.31656	1.09112	0.36129	0	Uiso	1
C14	C	0.35008	1.05696	0.35411	0	Uiso	1
C15	C	0.38633	1.07331	0.27503	0	Uiso	1
C16	C	0.61799	1.11097	0.21925	0	Uiso	1
C17	C	0.65377	1.14058	0.23377	0	Uiso	1
C18	C	0.68781	1.11861	0.3177	0	Uiso	1
C19	C	0.68489	1.06704	0.3905	0	Uiso	1
C20	C	0.64881	1.03802	0.37785	0	Uiso	1
C21	C	0.61514	1.05895	0.28931	0	Uiso	1
C22	C	0.64687	0.94768	0.14573	0	Uiso	1
C23	C	0.68038	0.91352	0.1386	0	Uiso	1
C24	C	0.67809	0.86135	0.20718	0	Uiso	1
C25	C	0.64135	0.8439	0.28071	0	Uiso	1
C26	C	0.60806	0.87862	0.28988	0	Uiso	1
C27	C	0.61061	0.93133	0.22479	0	Uiso	1
C28	C	0.34813	0.96661	0.122	0	Uiso	1
C29	C	0.31205	0.93759	0.1094	0	Uiso	1
C30	C	0.30913	0.88602	0.18222	0	Uiso	1
C31	C	0.34318	0.86406	0.26613	0	Uiso	1
C32	C	0.37895	0.89367	0.2806	0	Uiso	1
C33	C	0.38181	0.94569	0.2105	0	Uiso	1
C34	C	0.76188	1.12433	0.38828	0	Uiso	1
C35	C	0.79856	1.15237	0.39016	0	Uiso	1
C36	C	0.80031	1.2053	0.3296	0	Uiso	1
C37	C	0.76483	1.23021	0.26827	0	Uiso	1
C38	C	0.7282	1.20219	0.26638	0	Uiso	1
C39	C	0.72635	1.14888	0.32578	0	Uiso	1

C40	C	0.71049	0.77023	0.22375	0	Uiso	1
C41	C	0.74497	0.73744	0.22743	0	Uiso	1
C42	C	0.78371	0.7601	0.21654	0	Uiso	1
C43	C	0.78757	0.816	0.19897	0	Uiso	1
C44	C	0.75317	0.84862	0.19359	0	Uiso	1
C45	C	0.71427	0.82616	0.20676	0	Uiso	1
C46	C	0.24377	1.15601	0.3064	0	Uiso	1
C47	C	0.20937	1.18863	0.30107	0	Uiso	1
C48	C	0.21324	1.24453	0.28352	0	Uiso	1
C49	C	0.25197	1.2672	0.2726	0	Uiso	1
C50	C	0.28646	1.23441	0.27623	0	Uiso	1
C51	C	0.28267	1.17848	0.2932	0	Uiso	1
C52	C	0.26874	0.80245	0.23363	0	Uiso	1
C53	C	0.23212	0.77443	0.23178	0	Uiso	1
C54	C	0.19663	0.79934	0.17048	0	Uiso	1
C55	C	0.19838	0.85227	0.10989	0	Uiso	1
C56	C	0.23506	0.8803	0.11171	0	Uiso	1
C57	C	0.27059	0.85575	0.1742	0	Uiso	1
N58	N	0.8385	1.23252	0.32856	0	Uiso	1
C59	C	0.84305	1.28038	0.26464	0	Uiso	1
C60	C	0.88601	1.35767	0.18658	0	Uiso	1
C61	C	0.92348	1.38366	0.18179	0	Uiso	1
C62	C	0.95815	1.35975	0.25581	0	Uiso	1
C63	C	0.95422	1.30889	0.33427	0	Uiso	1
C64	C	0.91686	1.28256	0.3374	0	Uiso	1
C65	C	0.88259	1.30702	0.26381	0	Uiso	1
N66	N	0.17829	1.27873	0.27697	0	Uiso	1
N67	N	0.81866	0.72591	0.22312	0	Uiso	1
N68	N	0.15844	0.77212	0.17155	0	Uiso	1
N69	N	0.99703	1.38714	0.25154	0	Uiso	1
O70	O	0.46519	1.10222	0.36334	0	Uiso	1
O71	O	0.4598	0.90038	0.22499	0	Uiso	1
O72	O	0.53715	1.10427	0.2746	0	Uiso	1
O73	O	0.53176	0.90243	0.13634	0	Uiso	1
C74	C	0.85457	0.74449	0.25663	0	Uiso	1
C75	C	0.89072	0.71023	0.25838	0	Uiso	1
C76	C	0.92677	0.73141	0.32494	0	Uiso	1
C77	C	0.96216	0.7007	0.32444	0	Uiso	1
C78	C	0.9625	0.6484	0.25483	0	Uiso	1
C79	C	0.92595	0.62735	0.18852	0	Uiso	1
C80	C	0.89028	0.65784	0.19102	0	Uiso	1

N81	N	0.99991	0.6175	0.24867	0	Uiso	1
C82	C	0.14237	1.26015	0.24348	0	Uiso	1
C83	C	0.10622	1.29441	0.24176	0	Uiso	1
C84	C	0.15389	0.72426	0.2355	0	Uiso	1
C85	C	0.11436	0.69762	0.23635	0	Uiso	1
C86	C	0.07017	1.27323	0.17522	0	Uiso	1
C87	C	0.03478	1.30394	0.17573	0	Uiso	1
C88	C	0.03444	1.35624	0.24536	0	Uiso	1
C89	C	0.07099	1.37729	0.31165	0	Uiso	1
C90	C	0.10666	1.3468	0.30914	0	Uiso	1
C91	C	0.11094	0.64698	0.3136	0	Uiso	1
C92	C	0.07346	0.62099	0.3184	0	Uiso	1
C93	C	0.03879	0.64489	0.24438	0	Uiso	1
C94	C	0.04272	0.69575	0.16592	0	Uiso	1
C95	C	0.08008	0.72208	0.16277	0	Uiso	1
C96	C	0.99887	0.55922	0.24793	0	Uiso	1
C97	C	0.96753	0.53117	0.33236	0	Uiso	1
C98	C	0.0298	0.52951	0.16581	0	Uiso	1
C99	C	0.02942	1.47347	0.16786	0	Uiso	1
C100	C	0.99807	1.44542	0.25228	0	Uiso	1
C101	C	0.96715	1.47513	0.3344	0	Uiso	1
H102	H	0.41856	1.14039	0.14905	0	Uiso	1
H103	H	0.35821	1.2035	0.16674	0	Uiso	1
H104	H	0.28716	1.07629	0.42353	0	Uiso	1
H105	H	0.34817	1.01496	0.4133	0	Uiso	1
H106	H	0.59054	1.12899	0.14969	0	Uiso	1
H107	H	0.65546	1.18293	0.17718	0	Uiso	1
H108	H	0.7121	1.04856	0.46057	0	Uiso	1
H109	H	0.64641	0.99651	0.44028	0	Uiso	1
H110	H	0.64878	0.98967	0.08652	0	Uiso	1
H111	H	0.70992	0.92798	0.07664	0	Uiso	1
H112	H	0.63849	0.8012	0.33345	0	Uiso	1
H113	H	0.57837	0.86426	0.35077	0	Uiso	1
H114	H	0.35052	1.00812	0.05953	0	Uiso	1
H115	H	0.28471	0.95581	0.03954	0	Uiso	1
H116	H	0.34175	0.82175	0.32311	0	Uiso	1
H117	H	0.40641	0.87566	0.35015	0	Uiso	1
H118	H	0.7606	1.08114	0.43778	0	Uiso	1
H119	H	0.82723	1.13207	0.44124	0	Uiso	1
H120	H	0.76587	1.27352	0.21975	0	Uiso	1
H121	H	0.69947	1.22258	0.21631	0	Uiso	1

H122	H	0.67902	0.75156	0.2346	0	Uiso	1
H123	H	0.74156	0.69203	0.23935	0	Uiso	1
H124	H	0.81894	0.835	0.18899	0	Uiso	1
H125	H	0.75654	0.89395	0.17838	0	Uiso	1
H126	H	0.24041	1.11068	0.32159	0	Uiso	1
H127	H	0.1779	1.16992	0.31099	0	Uiso	1
H128	H	0.25559	1.31258	0.26068	0	Uiso	1
H129	H	0.31792	1.25308	0.26537	0	Uiso	1
H130	H	0.29747	0.78207	0.28368	0	Uiso	1
H131	H	0.23092	0.73112	0.28019	0	Uiso	1
H132	H	0.16979	0.87274	0.05869	0	Uiso	1
H133	H	0.23633	0.92349	0.0622	0	Uiso	1
H134	H	0.81589	1.30268	0.20735	0	Uiso	1
H135	H	0.85812	1.37781	0.12732	0	Uiso	1
H136	H	0.92628	1.42483	0.11725	0	Uiso	1
H137	H	0.98175	1.28866	0.39633	0	Uiso	1
H138	H	0.91422	1.24108	0.39993	0	Uiso	1
H139	H	0.8589	0.78901	0.2862	0	Uiso	1
H140	H	0.92723	0.77401	0.37988	0	Uiso	1
H141	H	0.99141	0.71795	0.3811	0	Uiso	1
H142	H	0.92513	0.58496	0.13186	0	Uiso	1
H143	H	0.86083	0.64005	0.1382	0	Uiso	1
H144	H	0.13804	1.21563	0.2139	0	Uiso	1
H145	H	0.18105	0.70196	0.29278	0	Uiso	1
H146	H	0.06971	1.23063	0.12026	0	Uiso	1
H147	H	0.00553	1.28669	0.11908	0	Uiso	1
H148	H	0.07181	1.41967	0.36833	0	Uiso	1
H149	H	0.13611	1.36458	0.36194	0	Uiso	1
H150	H	0.13883	0.62684	0.37285	0	Uiso	1
H151	H	0.07066	0.57982	0.38295	0	Uiso	1
H152	H	0.01519	0.71598	0.10386	0	Uiso	1
H153	H	0.08272	0.76356	0.10024	0	Uiso	1
H154	H	0.94203	0.55416	0.40018	0	Uiso	1
H155	H	0.05545	0.55187	0.09684	0	Uiso	1
H156	H	0.05475	1.45004	0.10047	0	Uiso	1
H157	H	0.94125	1.45346	0.40401	0	Uiso	1
H158	H	0.49868	1.10575	0.32467	0	Uiso	1
H159	H	0.49328	0.9038	0.18632	0	Uiso	1
Ni160	Ni	0.49847	0.50232	0.74981	0	Uiso	1
N161	N	0.45542	0.45443	0.72936	0	Uiso	1
C162	C	0.41998	0.47751	0.729	0	Uiso	1

C163	C	0.42191	0.53647	0.75963	0	Uiso	1
N164	N	0.45853	0.55422	0.78442	0	Uiso	1
N165	N	0.53842	0.45043	0.71527	0	Uiso	1
C166	C	0.57503	0.46817	0.74013	0	Uiso	1
C167	C	0.57696	0.52713	0.77077	0	Uiso	1
N168	N	0.54153	0.55021	0.77033	0	Uiso	1
C169	C	0.38888	0.62602	0.70996	0	Uiso	1
C170	C	0.35559	0.66074	0.71918	0	Uiso	1
C171	C	0.31885	0.64329	0.79274	0	Uiso	1
C172	C	0.31656	0.59112	0.86129	0	Uiso	1
C173	C	0.35008	0.55696	0.85411	0	Uiso	1
C174	C	0.38633	0.57331	0.77503	0	Uiso	1
C175	C	0.61799	0.61097	0.71925	0	Uiso	1
C176	C	0.65377	0.64058	0.73377	0	Uiso	1
C177	C	0.68781	0.61861	0.8177	0	Uiso	1
C178	C	0.68489	0.56704	0.8905	0	Uiso	1
C179	C	0.64881	0.53802	0.87785	0	Uiso	1
C180	C	0.61514	0.55895	0.78931	0	Uiso	1
C181	C	0.64687	0.44768	0.64573	0	Uiso	1
C182	C	0.68038	0.41352	0.6386	0	Uiso	1
C183	C	0.67809	0.36135	0.70718	0	Uiso	1
C184	C	0.64135	0.3439	0.78071	0	Uiso	1
C185	C	0.60806	0.37862	0.78988	0	Uiso	1
C186	C	0.61061	0.43133	0.72479	0	Uiso	1
C187	C	0.34813	0.46661	0.622	0	Uiso	1
C188	C	0.31205	0.43759	0.6094	0	Uiso	1
C189	C	0.30913	0.38602	0.68222	0	Uiso	1
C190	C	0.34318	0.36406	0.76613	0	Uiso	1
C191	C	0.37895	0.39367	0.7806	0	Uiso	1
C192	C	0.38181	0.44569	0.7105	0	Uiso	1
C193	C	0.76188	0.62433	0.88828	0	Uiso	1
C194	C	0.79856	0.65237	0.89016	0	Uiso	1
C195	C	0.80031	0.7053	0.8296	0	Uiso	1
C196	C	0.76483	0.73021	0.76827	0	Uiso	1
C197	C	0.7282	0.70219	0.76638	0	Uiso	1
C198	C	0.72635	0.64888	0.82578	0	Uiso	1
C199	C	0.71049	0.27023	0.72375	0	Uiso	1
C200	C	0.74497	0.23744	0.72743	0	Uiso	1
C201	C	0.78371	0.2601	0.71654	0	Uiso	1
C202	C	0.78757	0.316	0.69897	0	Uiso	1
C203	C	0.75317	0.34862	0.69359	0	Uiso	1

C204	C	0.71427	0.32616	0.70676	0	Uiso	1
C205	C	0.24377	0.65601	0.8064	0	Uiso	1
C206	C	0.20937	0.68863	0.80107	0	Uiso	1
C207	C	0.21324	0.74453	0.78352	0	Uiso	1
C208	C	0.25197	0.7672	0.7726	0	Uiso	1
C209	C	0.28646	0.73441	0.77623	0	Uiso	1
C210	C	0.28267	0.67848	0.7932	0	Uiso	1
C211	C	0.26874	0.30245	0.73363	0	Uiso	1
C212	C	0.23212	0.27443	0.73178	0	Uiso	1
C213	C	0.19663	0.29934	0.67048	0	Uiso	1
C214	C	0.19838	0.35227	0.60989	0	Uiso	1
C215	C	0.23506	0.3803	0.61171	0	Uiso	1
C216	C	0.27059	0.35575	0.6742	0	Uiso	1
N217	N	0.8385	0.73252	0.82856	0	Uiso	1
C218	C	0.84305	0.78038	0.76464	0	Uiso	1
C219	C	0.88601	0.85767	0.68658	0	Uiso	1
C220	C	0.92348	0.88366	0.68179	0	Uiso	1
C221	C	0.95815	0.85975	0.75581	0	Uiso	1
C222	C	0.95422	0.80889	0.83427	0	Uiso	1
C223	C	0.91686	0.78256	0.8374	0	Uiso	1
C224	C	0.88259	0.80702	0.76381	0	Uiso	1
N225	N	0.17829	0.77873	0.77697	0	Uiso	1
N226	N	0.81866	0.22591	0.72312	0	Uiso	1
N227	N	0.15844	0.27212	0.67155	0	Uiso	1
N228	N	0.99703	0.88714	0.75154	0	Uiso	1
O229	O	0.46519	0.60222	0.86334	0	Uiso	1
O230	O	0.4598	0.40038	0.72499	0	Uiso	1
O231	O	0.53715	0.60427	0.7746	0	Uiso	1
O232	O	0.53176	0.40243	0.63634	0	Uiso	1
C233	C	0.85457	0.24449	0.75663	0	Uiso	1
C234	C	0.89072	0.21023	0.75838	0	Uiso	1
C235	C	0.92677	0.23141	0.82494	0	Uiso	1
C236	C	0.96216	0.2007	0.82444	0	Uiso	1
C237	C	0.9625	0.1484	0.75483	0	Uiso	1
C238	C	0.92595	0.12735	0.68852	0	Uiso	1
C239	C	0.89028	0.15784	0.69102	0	Uiso	1
N240	N	0.99991	0.1175	0.74867	0	Uiso	1
C241	C	0.14237	0.76015	0.74348	0	Uiso	1
C242	C	0.10622	0.79441	0.74176	0	Uiso	1
C243	C	0.15389	0.22426	0.7355	0	Uiso	1
C244	C	0.11436	0.19762	0.73635	0	Uiso	1

C245	C	0.07017	0.77323	0.67522	0	Uiso	1
C246	C	0.03478	0.80394	0.67573	0	Uiso	1
C247	C	0.03444	0.85624	0.74536	0	Uiso	1
C248	C	0.07099	0.87729	0.81165	0	Uiso	1
C249	C	0.10666	0.8468	0.80914	0	Uiso	1
C250	C	0.11094	0.14698	0.8136	0	Uiso	1
C251	C	0.07346	0.12099	0.8184	0	Uiso	1
C252	C	0.03879	0.14489	0.74438	0	Uiso	1
C253	C	0.04272	0.19575	0.66592	0	Uiso	1
C254	C	0.08008	0.22208	0.66277	0	Uiso	1
C255	C	0.99887	0.05922	0.74793	0	Uiso	1
C256	C	0.96753	0.03117	0.83236	0	Uiso	1
C257	C	0.0298	0.02951	0.66581	0	Uiso	1
C258	C	0.02942	0.97347	0.66786	0	Uiso	1
C259	C	0.99807	0.94542	0.75228	0	Uiso	1
C260	C	0.96715	0.97513	0.8344	0	Uiso	1
H261	H	0.41856	0.64039	0.64905	0	Uiso	1
H262	H	0.35821	0.7035	0.66674	0	Uiso	1
H263	H	0.28716	0.57629	0.92353	0	Uiso	1
H264	H	0.34817	0.51496	0.9133	0	Uiso	1
H265	H	0.59054	0.62899	0.64969	0	Uiso	1
H266	H	0.65546	0.68293	0.67718	0	Uiso	1
H267	H	0.7121	0.54856	0.96057	0	Uiso	1
H268	H	0.64641	0.49651	0.94028	0	Uiso	1
H269	H	0.64878	0.48967	0.58652	0	Uiso	1
H270	H	0.70992	0.42798	0.57664	0	Uiso	1
H271	H	0.63849	0.3012	0.83345	0	Uiso	1
H272	H	0.57837	0.36426	0.85077	0	Uiso	1
H273	H	0.35052	0.50812	0.55953	0	Uiso	1
H274	H	0.28471	0.45581	0.53954	0	Uiso	1
H275	H	0.34175	0.32175	0.82311	0	Uiso	1
H276	H	0.40641	0.37566	0.85015	0	Uiso	1
H277	H	0.7606	0.58114	0.93778	0	Uiso	1
H278	H	0.82723	0.63207	0.94124	0	Uiso	1
H279	H	0.76587	0.77352	0.71975	0	Uiso	1
H280	H	0.69947	0.72258	0.71631	0	Uiso	1
H281	H	0.67902	0.25156	0.7346	0	Uiso	1
H282	H	0.74156	0.19203	0.73935	0	Uiso	1
H283	H	0.81894	0.335	0.68899	0	Uiso	1
H284	H	0.75654	0.39395	0.67838	0	Uiso	1
H285	H	0.24041	0.61068	0.82159	0	Uiso	1

H286	H	0.1779	0.66992	0.81099	0	Uiso	1
H287	H	0.25559	0.81258	0.76068	0	Uiso	1
H288	H	0.31792	0.75308	0.76537	0	Uiso	1
H289	H	0.29747	0.28207	0.78368	0	Uiso	1
H290	H	0.23092	0.23112	0.78019	0	Uiso	1
H291	H	0.16979	0.37274	0.55869	0	Uiso	1
H292	H	0.23633	0.42349	0.5622	0	Uiso	1
H293	H	0.81589	0.80268	0.70735	0	Uiso	1
H294	H	0.85812	0.87781	0.62732	0	Uiso	1
H295	H	0.92628	0.92483	0.61725	0	Uiso	1
H296	H	0.98175	0.78866	0.89633	0	Uiso	1
H297	H	0.91422	0.74108	0.89993	0	Uiso	1
H298	H	0.8589	0.28901	0.7862	0	Uiso	1
H299	H	0.92723	0.27401	0.87988	0	Uiso	1
H300	H	0.99141	0.21795	0.8811	0	Uiso	1
H301	H	0.92513	0.08496	0.63186	0	Uiso	1
H302	H	0.86083	0.14005	0.6382	0	Uiso	1
H303	H	0.13804	0.71563	0.7139	0	Uiso	1
H304	H	0.18105	0.20196	0.79278	0	Uiso	1
H305	H	0.06971	0.73063	0.62026	0	Uiso	1
H306	H	0.00553	0.78669	0.61908	0	Uiso	1
H307	H	0.07181	0.91967	0.86833	0	Uiso	1
H308	H	0.13611	0.86458	0.86194	0	Uiso	1
H309	H	0.13883	0.12684	0.87285	0	Uiso	1
H310	H	0.07066	0.07982	0.88295	0	Uiso	1
H311	H	0.01519	0.21598	0.60386	0	Uiso	1
H312	H	0.08272	0.26356	0.60024	0	Uiso	1
H313	H	0.94203	0.05416	0.90018	0	Uiso	1
H314	H	0.05545	0.05187	0.59684	0	Uiso	1
H315	H	0.05475	0.95004	0.60047	0	Uiso	1
H316	H	0.94125	0.95346	0.90401	0	Uiso	1
H317	H	0.49868	0.60575	0.82467	0	Uiso	1
H318	H	0.49328	0.4038	0.68632	0	Uiso	1

Table S5. Porous properties of COFs.

Sample	BET surface area (m^2 g^{-1})	Total pore volume (cm^3 g^{-1})	Average pore diameter (nm)
2D-Ni-N_4-COF	493	0.61	1.27
3D-Ni-N_4-COF	842	1.45	1.55

Table S6. Ni contents of the COFs.

Sample	ICP (wt.%) [a]	XPS (wt.%) [b]
2D-Ni-N_4-COF	2.4	4.3
3D-Ni-N_4-COF	3.1	5.0

[a] ICP is most accurate element counts for bulk sample.

[b] XPS is more sensitive to surface chemical composition.

Table S7. The Ni EXAFS curve-fitting parameters of COFs using the ARTEMIS module of IFEFFIT.

Sample	Shell	N	R (Å)	σ^2 (10^{-3}Å^2)	ΔE_0 (eV)	R factor
2D-Ni-N_4-COF	Ni-N	3.4±0.8	2.08±0.02	5.4±2.6	-1.5±2.5	0.0007
	Ni-Ni	2	3.09±0.01	3.8±0.8		
	Ni-C	4.4±1.1	3.52±0.06	3.0		
3D-Ni-N_4-COF	Ni-N	3.7±0.1	2.00±0.00	10.0	-7.5±0.5	0.0005
	Ni-Ni	2	3.09±0.00	3.8±0.3		
	Ni-C	7.3±0.8	3.37±0.01	9.0		

N, coordination numbers; R, the internal atomic distance; σ^2, Debye-Waller factor; ΔE_0, the edge-energy shift.

Table S8. The Ni EXAFS curve-fitting parameters of 2D-Ni-N_4-COF in situ experiment using the ARTEMIS module of IFEFFIT.

States	Shell	N	R (Å)	σ^2 (10^{-3}Å^2)	ΔE_0 (eV)	R factor
At 0.6 V for initial	Ni-N	3.7±0.3	2.09±0.00	6.0	2.8±0.5	0.01
	Ni-C	3.8±0.6	3.09±0.02	9.0		
	Ni-Ni	2.0±0.1	3.11±0.00	3.0		
At 0.6 V for reacting 20 min	Ni-N	4.4±0.3	2.09±0.00	7.2	-4.4±0.6	0.0006
	Ni-C	1.3±0.7	2.86±0.05	10.0		
	Ni-Ni	2.5±0.2	3.10±0.00	3.0		

N, coordination numbers; R, the internal atomic distance; σ^2, Debye-Waller factor; ΔE_0, the edge-energy shift.

Table S9. The Ni EXAFS curve-fitting parameters of 3D-Ni-N_4-COF in situ experiment using the ARTEMIS module of IFEFFIT.

States	Shell	N	R (Å)	σ^2 (10^{-3}Å^2)	ΔE_0 (eV)	R factor
At 0.6 V for initial	Ni-N	4.2±0.3	2.05±0.00	9.1±0.0	-6.0±0.2	0.0006
	Ni-C	2.7±0.2	2.96±0.00	9.0		
	Ni-Ni	2.1±0.0	3.09±0.00	3.0		
At 0.6 V for reacting 20 min	Ni-N	5.0±0.3	1.99±0.00	5.6	-11.0±0.6	0.005
	Ni-C	8.8±1.0	2.89±0.01	7.9		
	Ni-Ni	2.8±0.1	3.06±0.00	3.0		

N, coordination numbers; R, the internal atomic distance; σ^2, Debye-Waller factor; ΔE_0, the edge-energy shift.

Table S10. Calculation parameters of Ni-N_4 model.

	E(eV)	ZPE(eV)	TS(eV)		△E	△G
*	-909.6005075					
*-NO_3	-934.0322987	0.382867	0.267727	-933.9171587		-1.11910381
*-NO_3H	-938.3838378	0.700413	0.261584	-937.9450088	-1.10369881	-2.85375431
*-NO_2	-928.48288	0.24561	0.221612	-928.458882		3.37557232
*-NO_2H	-932.5807952	0.540561	0.169386	-932.2096202		-2.5766424
*-NO	-922.3216164	0.152664	0.139149	-922.3081014		3.79096436
*-HNO	-925.8745274	0.437066	0.214406	-925.6518674		-2.1696702
*-H_2NO	-929.9078533	0.781726	0.22504	-929.3511673		-2.52520403
*-H_2NOH	-934.2739801	1.118561	0.249878	-933.4052971		-2.88003406
*-H_2N	-924.1206637	0.64196	0.141026	-923.6197297		3.67501298
*-H_3N	-929.5462536	1.006691	0.18728	-928.7268426		-3.93301715

Table S11. Calculation parameters of Ni N_4-NO model.

	E(eV)	ZPE(eV)	TS(eV)		△E	△G
*	-922.3255014					
*-NO_3	-947.388443	0.400341	0.291824	-947.279926		-1.75687715
*-NO_3H	-951.1343128	0.700413	0.261584	-950.6954838		-2.24146199
*-NO_2	-941.6224786	0.389961	0.116318	-941.3488356		3.23609372
*-NO_2H	-945.3418255	0.529079	0.172941	-944.9856875		-2.46275612
*-NO	-935.0242272	0.13059	0.198648	-935.0922852		3.78284785
*-HNO	-938.712236	0.427095	0.208177	-938.493318		-2.22693698
*-H_2NO	-942.9030235	0.802909	0.197788	-942.2979025		-2.63048875
*-H_2NOH	-946.9084662	1.106632	0.204008	-946.0058422		-2.53384388
*-H_2N	-937.2411285	0.661992	0.119479	-936.6986155		3.19667231
*-H_3N	-942.1666647	1.000836	0.190206	-941.3560347		-3.48332346

References

[1] L. Sun, M. Lu, Z. Yang, Z. Yu, X. Su, Y.-Q. Lan, L. Chen, *Angew. Chem., Int. Ed.* 2022, **61**, e202204326.

[2] H. Yu, X. J. Wei, J. Li, S. Gu, S. Zhang, L. Wang, J. Ma, L. Li, Q.-z. Gao, R. Si, F. Sun, Y. Wang, F. Song, H. Xu, X.-h. Yu, Y. Zou, J. Q. Wang, Z. Jiang, Y. Y. Huang, *Nucl. Sci. Tech.* 2015, **26**, 050102-050101-050102-050107.

[3] M. Newville, *J. Synchrotron Radiat.* 2001, **8**, 322-324.
